# Using adjusted local assortativity with Molecular Pixelation unveils colocalization of membrane proteins with immunological significance

**DOI:** 10.3389/fimmu.2024.1309916

**Published:** 2024-06-25

**Authors:** Jan Rhomberg-Kauert, Max Karlsson, Divya Thiagarajan, Tomasz Kallas, Filip Karlsson, Simon Fredriksson, Johan Dahlberg, Alvaro Martinez Barrio

**Affiliations:** ^1^ Pixelgen Technologies AB, Stockholm, Sweden; ^2^ Department of Geodesy and Geoinformation, TU Wien, Vienna, Austria; ^3^ Department of Protein Science, Royal Institute of Technology, Stockholm, Sweden

**Keywords:** molecular pixelation, single cell, spatial proteomics, graph theory, topological data analysis, local assortativity, uropod formation, Rituximab

## Abstract

Advances in spatial proteomics and protein colocalization are a driving force in the understanding of cellular mechanisms and their influence on biological processes. New methods in the field of spatial proteomics call for the development of algorithms and open up new avenues of research. The newly introduced Molecular Pixelation (MPX) provides spatial information on surface proteins and their relationship with each other in single cells. This allows for *in silico* representation of neighborhoods of membrane proteins as graphs. In order to analyze this new data modality, we adapted local assortativity in networks of MPX single-cell graphs and created a method that is able to capture detailed information on the spatial relationships of proteins. The introduced method can evaluate the pairwise colocalization of proteins and access higher-order similarity to investigate the colocalization of multiple proteins at the same time. We evaluated the method using publicly available MPX datasets where T cells were treated with a chemokine to study uropod formation. We demonstrate that adjusted local assortativity detects the effects of the stimuli at both single- and multiple-marker levels, which enhances our understanding of the uropod formation. We also applied our method to treating cancerous B-cell lines using a therapeutic antibody. With the adjusted local assortativity, we recapitulated the effect of rituximab on the polarity of CD20. Our computational method together with MPX improves our understanding of not only the formation of cell polarity and protein colocalization under stimuli but also advancing the overall insight into immune reaction and reorganization of cell surface proteins, which in turn allows the design of novel therapies. We foresee its applicability to other types of biological spatial data when represented as undirected graphs.

## Introduction

The spatial organization of proteins governs a number of complex cellular processes such as cell signaling, cell–cell communication, and mobility. To enable the detection of proteins in cells and tissues, affinity reagents have remained the mainstay in the field. They have been used extensively in fluorescence microscopy tagged with fluorophores, typically providing fluorescence intensity data from each channel in one focal plane. The generation of three-dimensional information at high throughput and multiplexity is thus limited by the need for microscopy imaging. Imaging flow cytometry overcomes this throughput limitation by coupling traditional flow cytometers with the acquisition of an image of each cell (1). Fluorescence resonance energy transfer (FRET) microscopy measures the transfer of energy from an excited molecular fluorophore (the donor) to another fluorophore (the acceptor) (2). FRET microscopy imaging can achieve colocalization of labeled pairs of probes within sub-micron distances. However, the limitation of all microscopy techniques in terms of dimensionality and high-plexity information remains, as only a few antibodies can be acquired at the same time on the different microscope channels. Furthermore, the signal to noise is also hampered by auto-fluorescence, detector noise, optical noise, and spectral bleed-through between channels. Super-resolution imaging methods have provided groundbreaking insights in three-dimensional (3D) but are yet limited in multiplexing and throughput (3). Furthermore, super-resolution instrumentation is expensive and requires advanced training to even analyze the data.

To overcome the multiplexity problems, mass cytometry coupled antibodies to isotopes of different atomic weights that are detected by a mass spectrometer, such that the quantity of detected ions in a particular mass channel becomes a proxy for molecular detection (4). Although imaging mass cytometry (IMC) has been used with success in tissues, still, the multiplexity reported is still as high as 80 proteins (5). However, the application of IMC to tissues of 1-mm thickness (6) holds promise for 3D resolution on single cells one day. With the advent of next-generation sequencing (NGS), the tagging of antibodies with DNA oligos coupled to NGS readout has the potential of unlimited multiplexing. Although reading protein tags does not provide any spatial information (7), it has been shown to multiplex to 273 proteins (8). Other methods with different tagging strategies have been successful in showing antibody specificity to antigens by sequencing paired B-cell receptor (BCR) clonotypes interacting with DNA-barcoded antigens (9) or a recent proximity ligation assay by sequencing that is able to infer protein complexes (10). Similar to FRET, the drawback of the Prox-seq approach is that only proteins of interest are found in pairs, not larger constellations, and the location of proteins in the cell membrane is not achieved, as it lacks the relationship context.

As a result, developing a novel method to study spatial protein organization in a single cell has gained enormous significance in the past decade (11–13). A new emerging NGS-based method, Molecular Pixelation (MPX), provides spatial information on surface protein abundance and their relationship with each other on single cells in a three-dimensional field of view. Every single cell in MPX high-throughput datasets is encoded as a bipartite graph, which in turn can be analyzed to gain new insights into the colocalization of cell surface proteins (14). As graph metrics are commonly employed in social and biological networks, there are many analysis methods with potential applications for this new type of single-cell proteomics input data. In this study, we have adapted the application of local assortativity (15) to not only compare sets of proteins per node in the graph of each single cell but also numerically compare all nodes of the graph in terms of attribute distribution. Although MPX is not able to infer direct protein interactions in its current state, it enables the discovery of protein constellations of biological significance and allows the exploration of protein colocalization as a novel therapeutic target.

## Materials and methods

### Molecular Pixelation

The MPX workflow builds an amplicon in three steps: the first step involves staining the cells with antibody–oligonucleotide conjugates (AOCs). In the next step, a set of DNA pixels, each containing a unique sequence identifier so-called A-pixel, hybridize into a group of spatially proximal AOCs each, and a gap-fill ligation reaction adds the unique sequence identifier to the AOC, imprinting AOCs with the same A-pixel neighborhood tag. Next, a second reaction is performed with a set of B-pixels connecting several A-pixel areas. The combined spatial information imprinted by A- and B-pixels preserves the information of which protein molecules were spatially adjacent on the original cell surface (14).

MPX data from any immune cell in solution can be represented as a bipartite graph G, where A- and B-pixels are nodes interconnected by a set of AOCs as edges. We transformed each bipartite graph G into an A-node projection, where edge attributes of the bipartite graph become node attributes of the projected graph in the A-node of the A–B parts (14). Subsequently, the A-nodes become directly connected following the original connections of the B-nodes. We used A-node projected graphs from the original bipartite graph G throughout this study to move the antibody edge labels and counts into A-nodes and to be able to use local assortativity. Local assortativity only works for MPX if protein labels and counts are projected to the A-node. For the rest of this study, when we used the concept of node or vertex on a cell graph, and we referred to an A-node with antibody labels and counts.

MPX can record the counts of each protein molecule, which can be used to assess differences in protein abundance between cell states or conditions. However, the two most important features of this data type are to be able to study the relative positioning of individual protein markers, as well as their colocalization. First, the Jaccard Index and Pearson’s correlation across different proteins in the same single-cell graph were used in order to ascertain if two proteins tend to colocalize or not upon stimulation. MPX global measure to study homophily/heterophily in single-cell graphs currently requires the definition of a local neighborhood parameter to identify molecules present in pixels assigned to a given antibody (14), and it would be desirable to have a parameter-free definition of local neighborhoods.

#### Molecular Pixelation datasets

Karlsson and colleagues, by applying the MPX workflow, generated several datasets^1^ demonstrating the technological capabilities of MPX for different applications (14).

**Figure 1:**
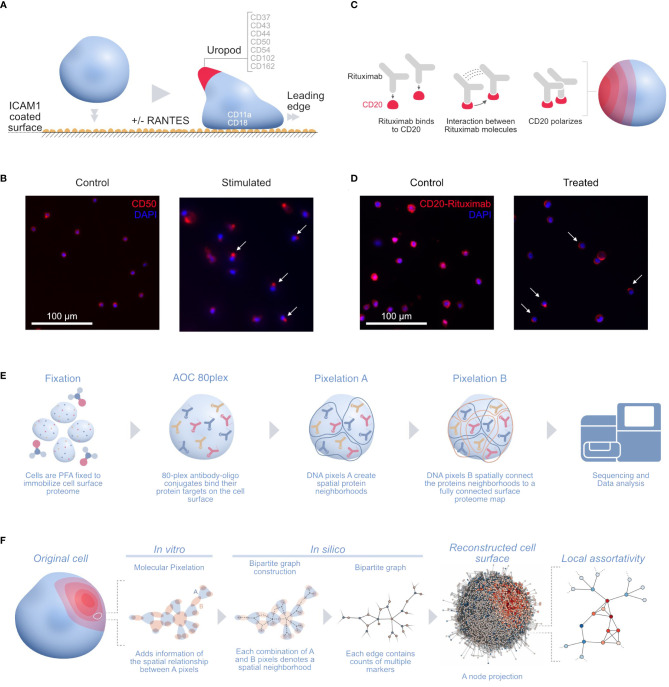
**(A)** Illustration of the uropod formation on the CD54 coated surface and proteins previously associated with T-cell uropods: ICAMs (ICAM1–3: CD54, CD102, and CD50), mucins (CD43 and CD162), and integrins (CD11a/CD18; αLβ2 integrin or LFA-1) (16); CD44 (19, 20); and CD37 (21, 22). **(B)** Widefield immunofluorescence microscopy picture of the uropod formation in both control (left) and stimulated cells (right) with CD50 (red, phycoerythrin fluorophore) and stained nuclei (blue, DAPI). Some of the polarized CD50 pertaining to the uropods upon stimulation are marked by white arrows in the picture. **(C)** Illustration of the stimulated CD20 receptors on the RTX-treated B-cell sample. Here, the RTX monoclonal antibodies interact with each other, thus creating a strong polarization cap on one side of the cell. **(D)** Widefield immunofluorescence microscopy picture of the RTX experiment with control (left) and treated (right) Raji cells with CD20-RTX (red, phycoerythrin fluorophore) and stained nuclei (blue, DAPI). Cells polarized after RTX capping are marked by white arrows in the picture. **(E)** The MPX workflow starts with cell fixation to immobilize the proteome on the surface of the cell, followed by staining with the AOC panel and two steps of Molecular Pixelation before a library is prepared for sequencing. **(F)** Illustration of the cell-to-graph transformation as explained in the MPX study (14). The double MPX workflow step carried out *in vitro* denotes a spatial neighborhood represented by a bipartite graph with AOC molecule counts associated with the edges. This bipartite graph can then be represented into its A-nodes. The A-node projection results in a shift of information from the edges to the vertices. Based on these vertex attributes, we can now compute the (adjusted) local assortativity for each vertex and color the nodes on a scale from assortative (red) to uniform mixing (white) to disassortative (blue), as seen in the last step of the panel (15). ICAMs, intercellular adhesion molecules; RTX, Rituximab; MPX, Molecular Pixelation; AOC, antibody–oligonucleotide conjugate.

One of those applications is stimulating human T cells with phytohemagglutinin (PHA) followed by IL2 for 5 days into the formation of uropods. Leukocyte migration prompts the formation of distinct structures in cells in order to follow chemotactic gradients and reach the target tissue. Leukocytes polarize and convert mechanical force into forward locomotion by coordinating a regulated bidirectional cycle: the leading edge pushes the cell forward, whereas the plasma membrane moves to the rear (16). The leukocyte uropod formation was first described during studies of the interactions between T lymphoblasts and macrophages (17). Irrespective of the cell type, the uropod trailing protrusion, referred to as the “uropod knob” (18), involves intracellular actin polymerization and actomyosin contraction providing the force that creates the protrusion. CD50 (ICAM3) and several proteins are supposed to colocalize on the uropod structure (**Figure 1A**) (19) with CD50 polarization being validated by microscopy (**Figure 1B**) (14).

Karlsson and colleagues fixed and cultured PHA blasts on plates coated with either 5 µg/mL of CD54Fc antibody alone or with two different chemotactic cytokines in solution or 10 ng/mL of CCL5 (RANTES) in one condition or CCL2 (MCP1) in another at 37°C for 1 h. We downloaded the output dataset PXL files from three of the conditions in the experiment^2^, one with cells fixed with CD54 and stimulated within solution RANTES (“uropod CD54 fixed RANTES stimulated”, 657 cells), a second one with cells fixed with CD54 (“uropod CD54 fixed”, 733 cells), and the last with cells in solution without the stimulation as a control (“uropod control”, 658 cells); for the rest of this paper, we will refer to these datasets as stimulated cells, fixed control, and control, respectively.

In another MPX application, Raji cells (ATCC, Manassas, VA, USA)^3^ were Fc-receptor blocked with 50 µg/mL of human IgG for 15 min at 4°C and washed. Cells were then either fixed directly with paraformaldehyde (PFA) (“control”, 607 cells) or incubated with 20 µg/mL of rituximab (RTX) (ProteoGenix, Schiltigheim, France) with a specific AOC (“treated”, 873 cells) in Roswell Park Memorial Institute (RPMI) media for 60 min at 37°C, followed by PFA fixation and washing. RTX, a monoclonal antibody therapy approved for medical use in 1997, targets CD20 primarily on the surface of B cells. RTX mediates antibody-dependent cellular cytotoxicity (ADCC), allowing specific NK-cell killing (23) (**Figure 1C**) by polarizing CD20 on a cap at the surface of B cells (**Figure 1D**).

We downloaded the output dataset PXL files from the two conditions in the Raji cell MPX experiment^4^ (**Figure 1E**) and applied adjusted local assortativity to them. Comparing both populations of treated and control cells allowed us to recapitulate the already described RTX mechanism of action (23).

### Local assortativity

Assortativity is a well-known concept in graph theory and network science, which compares the patterns of vertex attributes across the network. The most common version is the global assortativity where the whole graph is considered and the vertices are compared on a global scale (24, 25). A downside of the global measurement is that it does not account for local heterogeneity in subregions of the network. This problem was overcome by different versions of the local assortativity, which focused on studying the homogeneity in communities of labeled networks. The advantage of the local assortativity is that each vertex gets assigned a score based on the attribute of interest, and thus, one can analyze in detail the distribution of the network properties (15, 26, 27). A classic example of this is calculating the local assortativity for the degree of each vertex (**Figure 1F**). We transformed every cell bipartite graph into their A-node projected graph and transferred the labels from the edges to the vertices to be able to apply local assortativity and calculate an assortativity score for each vertex.

**Figure 2:**
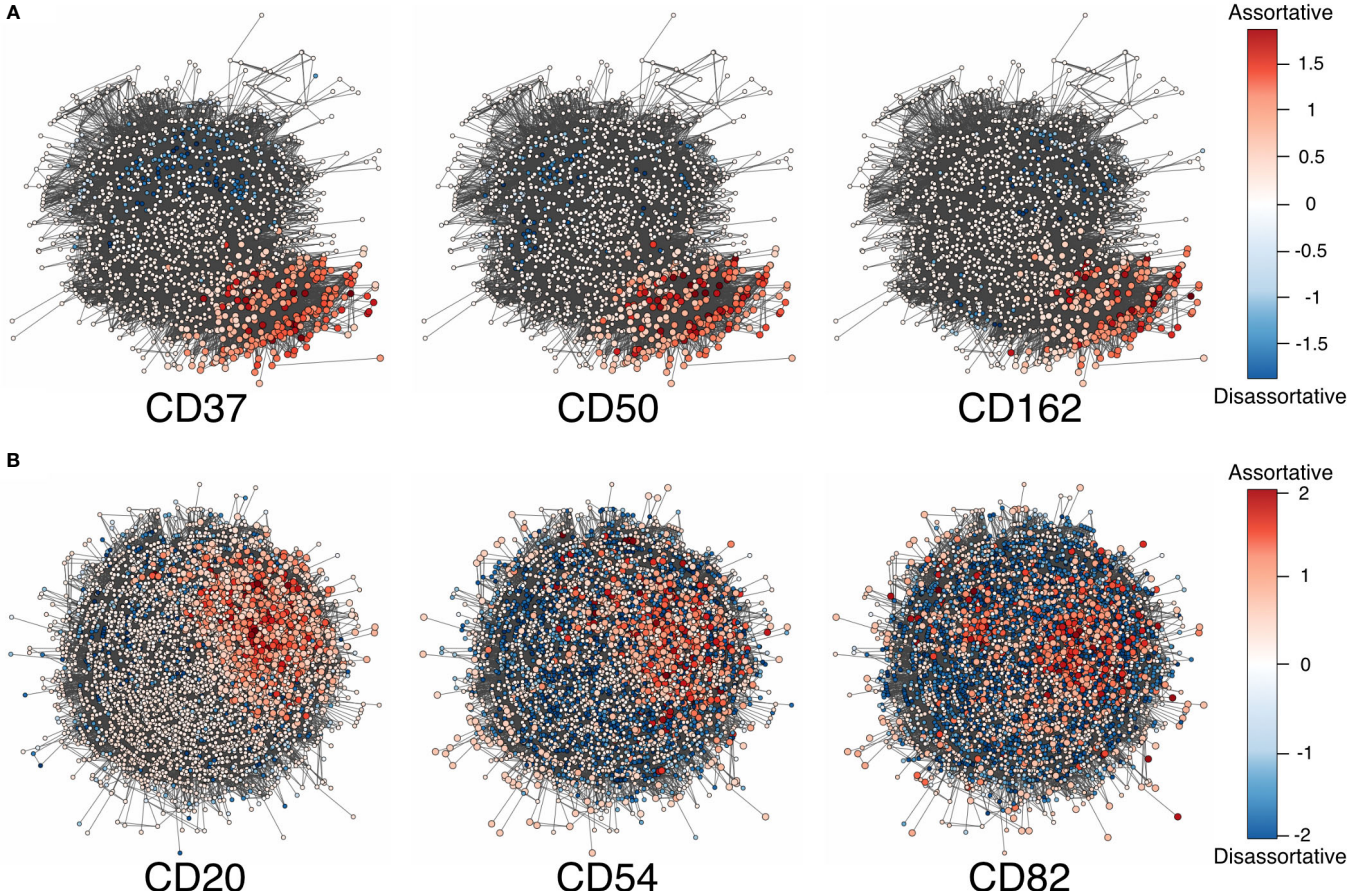
**(A)** Adjusted local assortativity scores for the CD50, CD162, and CD37 displaying the characteristic uropod in one of the cells from the stimulated chemotactic experiment. The color scheme here is a gradient from high local assortativity in red to high local disassortativity in blue with uniform mixing in white. **(B)** Stimulated cells from the RTX-treated experiment where CD20, CD54, and CD82 are colored by the adjusted local assortativity. RTX, Rituximab.

Herein, we used local assortativity as defined by Peel and colleagues (15) and applied it to two of the MPX public datasets (**Figure 2**) generated with slight modifications in the PageRank threshold (**Supplementary Data 1.1**).

In addition to the 76 antibodies targeting specific protein epitopes in the panel (14), three mouse isotype control antibodies were included (mIgG2b, mIgG1, and mIgG2a). With the information provided by these control proteins, we determined a lower boundary required in order to calculate the colocalization score on the other 76 proteins. This “isotype threshold” is set by the maximum number of isotype protein counts per cell based on the three mouse controls.


$$threshold_{proteins}=max(N_{proteins}(mIgG2b),N_{proteins}(mIgG1),N_{proteins}(mIgG2a))$$

If the number of raw molecule counts for a given protein in a cell is below that threshold, the assortativity scores are set to zero for that protein in all nodes of the A-node graph. A second filter (“vertex threshold”) will require each protein to be present in more than 10 vertices on the A-node graph. If those limits are not met, we consider that there is too little information in the cell to create a high-quality local assortativity distribution and all the A-nodes for that protein on that cell are also initialized to zero.

### Bound version of local assortativity

The original version of local assortativity defined by Peel and colleagues (15) did not have explicit boundaries, and the distribution of values was not generally comparable across different graphs. Therefore, in this study, we had to improve the score by creating an adjusted version, which improves the general comparison across graphs and values. With this in mind, first, we created a distribution that would have similar maximum and minimum values for the same marker when looking at different cells. These values would imply a boundary for each distribution, allowing us to compare the scores across cells and samples.

To account for the different scores across graphs, we adjusted local assortativity to have zero mean by reweighting the positive and negative scores separately and preserving zero as uniform mixing. This also has the advantage of creating a boundary in both directions. In practice, we therefore compute first the local assortativity as defined by Peel et al. and divide each positive value by the sum of all positive values to normalize the data. The same is done for the negatives by the sum of all negatives.

In mathematical notation, this is equivalent to the following statements.

Let *x_j_* be the unadjusted local assortativity scores as calculated by Peel et al. (15), and then normalized assortativity *f*(*x_j_*) is defined as

$f(x_{j})=\frac{x_{j}}{\sum {\;}_{i=1,x_{i}>0}^{n}\;x_{i}}$ for *x_j_* ≥ 0 and $\sum {\;}_{i=1}^{n}\;|x_{i}|>0$ ,

$f(x_{j})=\frac{x_{j}}{\sum {\;}_{i=1,x_{i}<0}^{n}\;|x_{i}|}$ for *x_j_* < 0 and $\sum {\;}_{i=1}^{n}\;|x_{i}|>0$

and *f*(*x_j_*) = 0 for and $\sum {\;}_{i=1}^{n}\;|x_{i}|=0$ .

After the first step of normalization, we adjust the created score to have one standard deviation dividing each value of the scores from the previous equation by the standard deviation of the distribution. This results in global upper and lower limits for the normalized local assortativity distribution. Rewriting now the first equation for simpler notation, we get $\tilde{x_{j}}=f(x_{j})$ , which when divided by the standard deviation gives us the normalized standardized local assortativity of the workflow $ɡ\;(\tilde{x_{j}})$ .

$ɡ\;(\tilde{x_{j}})=\frac{\tilde{x_{j}}}{\sum {\;}_{i=1}^{n}\;(\tilde{x_{i}}-\mu ){\;}^{2}}=\frac{\tilde{x_{j}}}{\sum {\;}_{i=1}^{n}\;{\tilde{x_{i}}}^{2}}$ for $\sum {\;}_{i=1}^{n}\;|\tilde{x_{i}}|>0$ ,

and $ɡ\;(\tilde{x_{j}})=0$ for $\sum {\;}_{i=1}^{n}\;|\tilde{x_{i}}|=0$ .

In order to correct for outliers and homogenize the scale of this distribution, akin to standard single-cell methods (28), we used the log transformation to create a more uniform distribution (**Supplementary Figure 2**). Here, we observed similar minima and maxima across multiple proteins, assuring the comparability of the scores across different cells or, more generally, different networks containing attribute information. Therefore, our work improves the previous local assortativity method (15) specifying the upper and lower bounds and enabling representative comparisons and aggregations.


$$h(z_{j})=\mathrm{sgn}(z_{j})\cdot log(|z_{j}|+1)$$

$$\Rightarrow \varrho (x_{j})=(h\circ g\circ f)(x_{j})$$

By combining all these transformations, we obtained the adjusted version of local assortativity, and proof for its bounds can be found in **Supplementary Data 1.2**.

### Pairwise colocalization

Our aim was to look at any combination of proteins colocalizing, but initially, we created a metric that outputs the colocalization of two proteins by combining the newly introduced adjusted local assortativity measurements. With local assortativity, we had positive and negative values for each node; thus, colocalization would translate to the correlation of vertex values. Therefore, we can apply Spearman’s correlation to create a metric that yields the desired colocalization for the two given proteins of interest.

Let *n* be the number of vertices in the graph, *x_i_* and *y_i_* be the local assortativity scores for two proteins on a vertex in the graph with 0 ≤ *i* ≤ *n*, and *R* the rank transformation. Then, the colocalization score of two proteins *X* = {*x*_0_,*x*_1_,…,*x_n_*} and *Y* = {*y*_0_,*y*_1_,…,*y_n_*}can be expressed using *ϱ*(*x*) and Spearman’s correlation (29–31) as


$$coloc(X,Y)=\frac{\sum {\;}_{i=1}^{n}\;R(\varrho (x_{i}))\cdot R(\varrho (y_{i}))}{\sqrt{\sum {\;}_{i=1}^{n}\;R{\left(\varrho (x_{i})\right)}^{2}\cdot \sum {\;}_{i=1}^{n}\;R{\left(\varrho (y_{i})\right)}^{2}}}$$

Proteins that failed to pass our filters (“isotype and vertex thresholds”) were zeroed for every node in the A-node graph of the cell. Additionally, in the special case that one of the two adjusted local assortativity scores was zero for every node, the pairwise colocalization score would be defined as zero to avoid edge cases with Spearman’s correlation. This zeroing in the colocalization measure was well aligned with the local assortativity distribution where random noise could be thought of as a case of uniform mixing.

Proteins measured with AOCs give a relative measurement per cell, making the pairwise local assortativity scores difficult to interpret in terms of absolute values. Therefore, a more robust approach is to compare among experimental conditions, i.e., the uropod-stimulated sample to the control sample. Therefore, we calculated differential colocalization by comparing the scores of the uropod-stimulated sample with both control samples, fixed and in solution. In the RTX experiment, the treated sample was compared to a corresponding control. All statistical tests were performed using Wilcoxon rank sum tests of different contrasts.

### Higher-order colocalization

We ultimately aimed to assess proteins that colocalize in groups larger than in pairs. However, we only performed pairwise protein comparisons at the moment. Therefore, a new kind of similarity measure is required to calculate colocalization for multiple proteins. In an ecosystem, multi-species interactions can be measured in multiple sites at the same time using specific scores (32). We adapted this measure to reflect the overlap of local assortativity regions and compare the colocalization of multiple proteins at the same time.

The multiple-site similarity measure (32) is defined as


$$\begin{array}{l} C_{s}^{\text{ T}}=\frac{T}{T-1}(\frac{\sum {\;}_{i<j}\;|A_{i}\cap A_{j}|-\sum {\;}_{i<j<k}\;|A_{i}\cap A_{j}\cap A_{k}|+\ldots +|A_{i}\cap \ldots \cap A_{T}|}{\sum {\;}_{i}\;|A_{i}|})\\ =\;\frac{T}{T-1}(1-\frac{\left|\cup_{i=1}^{T}\;A_{i}\right|}{\sum {\;}_{i=1}^{T}\;|A_{i}|}) \end{array}$$

In the simplest case, where *T* = 2, this simplifies the Sørensen similarity index (**Supplementary Figure 3A**) (32, 33).

In the cases where *T* ≥ 3, we can apply this similarity measure to protein colocalization of multiple proteins (**Supplementary Figure 3B**). The multiple-site similarity is well suited for hypothesis testing on the putative colocalization of a group of proteins, as the comparison of proteins increases factorially.

Here, the exact selection of the sets that should be compared was made based on the adjusted local assortativity scores. First, we selected a threshold for the set of nodes we wanted to compare as the scores are numerical values. Given that local assortativity was centered around zero, we decided to select a threshold centered in zero and proceeded with all values with a score greater than zero. Effectively, this means a selection on all the nodes displaying assortativity rather than a random distribution of proteins (i.e., uniform mixing).

## Results

### Pairwise colocalization

**Figure 3:**
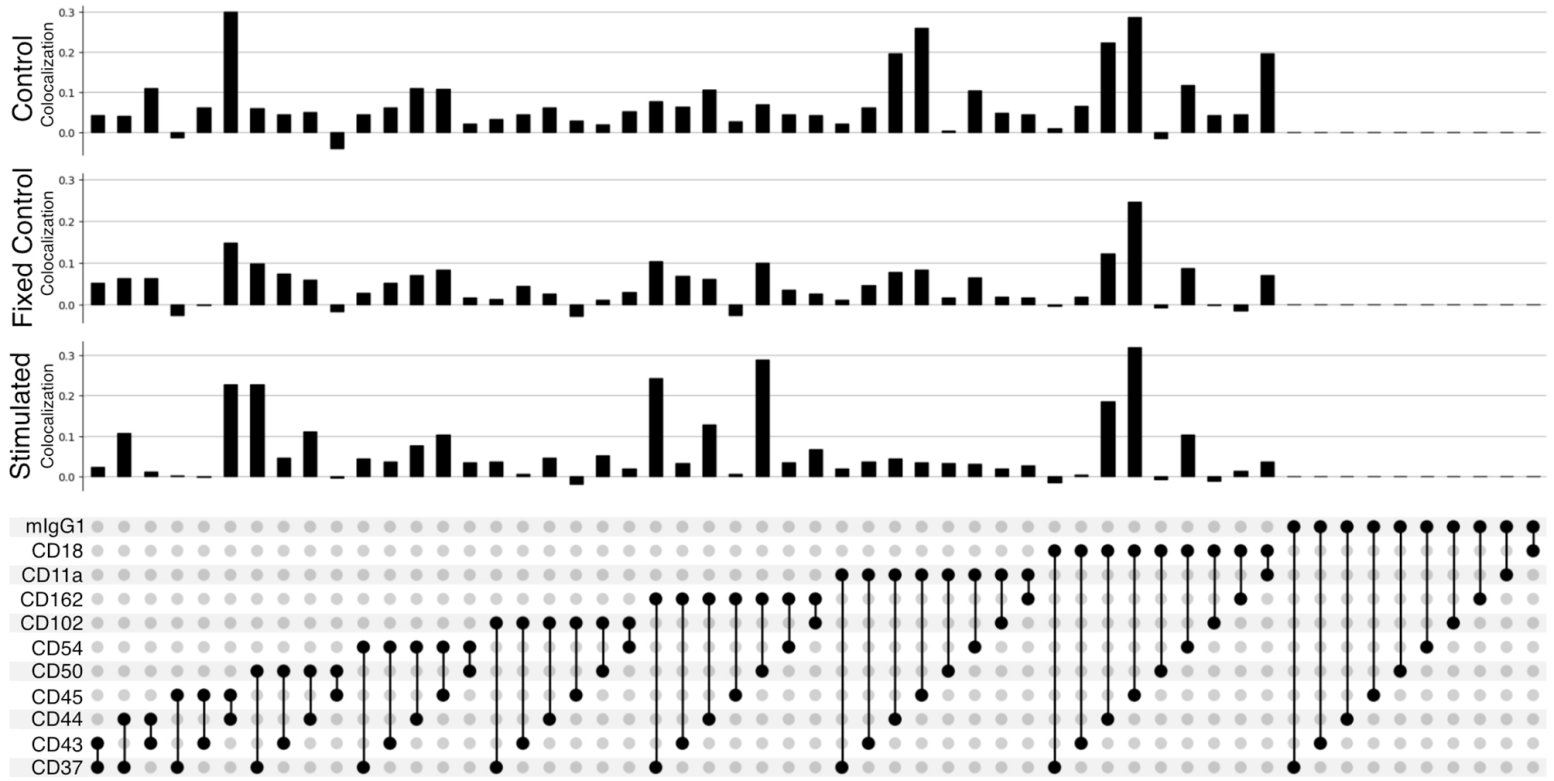
Pairwise colocalization of selected proteins shown by an UpSet plot (34). Each barplot represents the colocalization score of two proteins on the control, fixed control, and stimulated samples. The link panel at the bottom shows what pairs of proteins are interrogated in each respective barplot above.

The improved pairwise analysis of protein regions on single cells across the control, fixed control, and stimulated sample (**Figure 3**) replicates the results found by Karlson and colleagues (14) using different computational methods. Three proteins (CD162, CD37, and CD44) strongly colocalize with CD50 on the uropod (**Figure 3**). Specifically, we can observe that CD50, CD162 (P-selectin glycoprotein ligand 1 or PSGL1), and CD37, a member of the tetraspanin family, show the highest pairwise colocalization when stimulated with RANTES. The pair CD50 and CD162 is only second to CD45 and CD18 in the stimulated condition. Other known adhesion molecules such as CD102 (ICAM2) did not show a high colocalization with either of these proteins in the stimulated condition. We also noticed that some of the highly abundant protein pairs, such as CD18 and CD45, colocalized in all the conditions: control, fixed control, and stimulated cells (**Figure 3**). To account for biases toward highly abundant proteins, we assessed whether they could be overcome by employing permutation testing (**Supplementary Data 1.3** and **1.4**). However, the correction from permutation per vertex is on average less than 1% from the original adjusted score in important uropod proteins (CD50) at the expense of a much longer runtime. Therefore, in the end, we decided to omit permutation testing for the results presented in this paper.

When fixing cells, we expected to observe CD11a/CD18 complex (αLβ2 integrin or LFA-1) binding to the CD54Fc antibody coated in the plates. However, the pairwise colocalization, although present and uniformly mixed, is not as strong as expected (**Figure 3**).

Furthermore, the pairwise comparison of CD50 with the colocalization of one of the isotype controls (mIgG1) (**Figure 3**) can be interpreted as background noise level and shows the significance of the colocalization on pairwise combinations among CD50, CD162, and CD37.

### Pairwise differential colocalization

**Figure 4:**
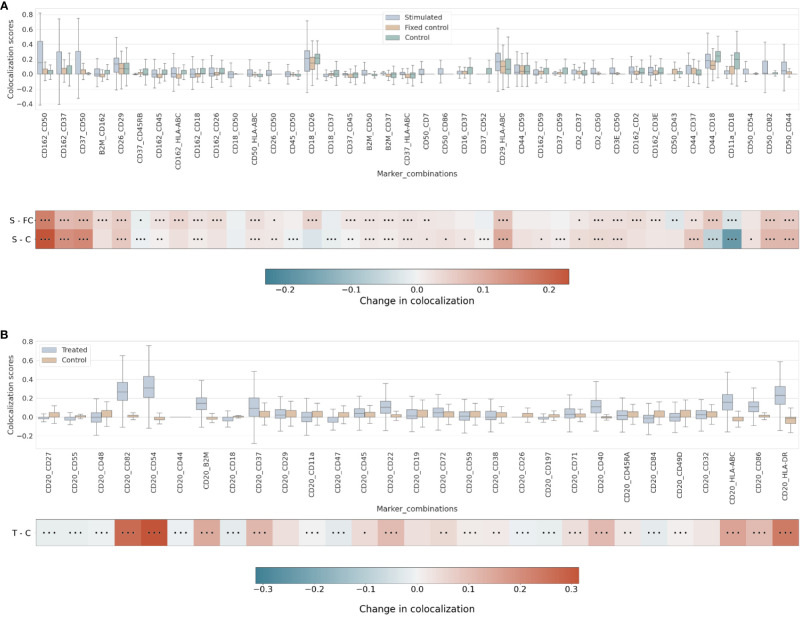
**(A)** Differential pairwise colocalization on a group of selected proteins compared for the three experimental conditions in the uropod experiment (see “Molecular Pixelation datasets” in the Materials and Methods section for a description). First, distributions of all pairwise values per cell are visualized as boxplots per condition side by side. Then, in the first row, differential colocalization between the stimulated (S) and fixed control (FC) samples is measured per pairwise comparison as mean(S) − mean(FC) scores per protein. In the second row, differential colocalization between the stimulated (S) and control (C) samples. The dots indicate p-value ranges generated by a Wilcoxon rank test: 0 dots (p-value > 0.01), 1 dot (0.001< p-value ≤ 0.01), 2 dots (0.0001< p-value ≤ 0.001), and 3 dots (p-value ≤ 0.0001). The scale bar of these differences is shown at the bottom. **(B)** Differential pairwise colocalization of CD20 with selected proteins from the treated (T) and control (C) samples of the RTX experiment. Analog to panel A, the differential colocalization is given by the mean difference of both samples, mean(T) − mean(C), and the dots indicate the same p-value changes as before. RTX, Rituximab.

Our differential colocalization analysis compares first the scores of the uropod-stimulated sample against the two control samples (**Figure 4A** and **Supplementary Figure 7**).

When comparing experimental conditions, a pronounced increase in colocalization of the uropod structural proteins in stimulated cells could be observed when compared to the unstimulated control cells, both fixed and in solution, that cannot be associated with experimental fixation (**Figure 4A**). This is especially striking in the colocalization scores in all pairwise comparisons of CD50, CD162, and CD37 (**Figure 4A**) (p-value ≤ 0.0001, Wilcoxon rank test). Otherwise, we observed significant differences (p-value ≤ 0.0001, Wilcoxon rank test) at that level in mean colocalization scores across the three experimental conditions involving one of those three proteins in the pair and highly abundant proteins (HLA-ABC, B2M, CD2, and CD3E). However, the mean difference to the control conditions was small in all those cases (<0.05). By taking CD82 or CD44 (P-glycoprotein 1) proteins combined with CD50, we observed a consistent difference in mean colocalization (>0.05) and very significant at the same time (p-value ≤ 0.0001, Wilcoxon rank test) (**Figure 4A**).

More interestingly, there were some proteins showing high colocalization with the same sign only in stimulated cells, such as CD26 and CD29 (**Figure 4A**), compared to the control condition samples (p-value ≤ 0.0001, Wilcoxon rank test). CD29 was also colocalized with HLA-ABC with a mean difference larger than 0.05. The only two proteins with such a significant difference and opposite signs against each contrast were CD18 and CD44.

RTX induces the capping of CD20 on the surface of B cells (35) (23). In our pairwise analysis with CD20, there was a strong increase of colocalization with CD54 (ICAM1) or CD82 when comparing treated and control conditions (**Supplementary Figure 8**). When compared to the control experiment, those two pair combinations showed a stronger signal than when comparing CD20 and other highly abundant proteins, such as HLA-DR or HLA-ABC/B2M with high significance (p-value ≤ 0.0001, Wilcoxon rank test) (**Figure 4B** and **Supplementary Figure 9**). CD82 is a membrane glycoprotein of the tetraspanin family found associated with both B-cell MHC class II compartments (36) and CD20 in supramolecular complexes (37). Other proteins found with slightly lower pairwise differential colocalization (>0.75) but high significance (p-value ≤ 0.0001, Wilcoxon rank test) were CD37, CD22, CD40, and CD86 (**Figure 4B** and **Supplementary Figure 9**).

### Higher-order colocalization

When assessing combinations in the stimulated condition of three proteins (i.e., trios), higher-order colocalization allows us to specifically test multiple proteins combined and their colocalization relationship in the same cell graph. Higher-order colocalization was applied to the adjusted local assortativity values of the different experiments and conditions and only calculated on a subset of proteins of interest from the pairwise results shown above in the uropod and RTX experiments.

On the uropod datasets, we observed the highest colocalization score on the stimulated sample among the trio comparison (order of 3) containing CD44, CD45, and CD162 (**Supplementary Figure 10**). Furthermore, we observed the second-highest colocalization on CD44, CD45, and CD54 (**Supplementary Figure 10**), which are also well-known uropod proteins (16). One of these proteins (CD44) is specifically involved in the uropod formation (16, 38), and another (CD45) is a widely abundant pan-lymphocyte signaling molecule. Furthermore, all combinations of order 3 containing two out of CD43, CD44, CD50, and CD54 produce high colocalization scores (>0.10) (**Supplementary Figure 10**).

**Figure 5:**
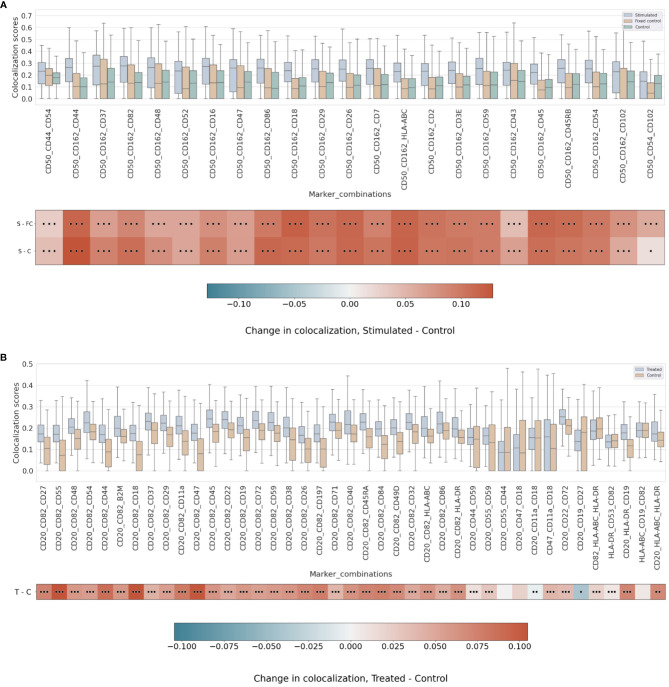
**(A)** Differential higher-order colocalization of trios (order 3) on a group of selected proteins for the three experimental conditions in the uropod experiment (see “Molecular Pixelation datasets” in the Materials and Methods section for a description). First, distributions of all protein comparison values per cell are visualized as boxplots per condition side by side. Then, in the first row, differential colocalization between the stimulated sample (S) and the fixed control (FC) is measured per pairwise comparison as mean(S) − mean(FC) scores. In the second row, differential colocalization between the stimulated (S) and control (C) samples can be found. The dots indicate p-value ranges generated by a Wilcoxon rank test: 0 dots (p-value > 0.01), 1 dot (0.001< p-value ≤ 0.01), 2 dots (0.0001< p-value ≤ 0.001), and 3 dots (p-value ≤ 0.0001). The scale bar of these differences is shown at the bottom. **(B)** Differential higher-order colocalization of CD20 and CD54 with different proteins of relevance on a trio (order 3). The boxplots display the two experimental conditions, RTX-treated (T) and control (C), and compare these by subtracting mean(T) − mean(C) for each protein distribution. The significance of the differences in the differential analysis was analogously computed to panel A using the Wilcoxon rank test and displaying significance using the same dot nomenclature as before. RTX, Rituximab.

Strikingly, the combination of CD50 and CD162 with CD44 has one of the highest scores of colocalization in the stimulated sample as well as the largest mean differential colocalization with the controls (**Figure 5A** and **Supplementary Figure 11**) on all trios displayed compared to CD50 and CD162 with CD37, which had the highest pairwise colocalization scores between them behind CD18 and CD45 (**Figure 3**). As expected, these proteins (CD50, CD162, CD44, and CD37) colocalized in the same cellular region (**Supplementary Figure 12**) and were in alignment with scientific knowledge about the uropod formations (19). However, the CD50, CD162, and CD44 trios demonstrated that our higher-order colocalization method was able to improve scoring even when pairs had shown lower pairwise colocalization scores than others.

In the RTX experiment, by combining pairwise scores into a higher order of 3, the scores of CD20, CD54, and CD82 were expected to be at the top. Surprisingly, our colocalization method for higher orders detects new trios with mean colocalization larger than the score of those three proteins. The combination of CD20 and CD82 with CD45, CD22, CD72, or CD37 in the treated sample produced a higher mean colocalization of order 3 with high significance (p-value ≤ 0.0001, Wilcoxon rank sum) (**Figure 5B** and **Supplementary Figure 13**). Interestingly, CD45 or CD37 pairwise colocalized with CD20 had both lower significance and mean differential colocalization to controls compared to CD54 or CD82 with CD20 (**Figure 4B**). However, when CD45 or CD37 was combined together with CD20 and CD82, they scored two of the 10 highest mean colocalization scores with very high significance (p-value ≤ 0.0001, Wilcoxon rank sum) (**Figure 5B**). Again, this is another observation that our method was able to improve scoring in cases of pairs with lower pairwise colocalization scores.

Other proteins of interest in the order of 3 that colocalized with CD20 and CD82, albeit with lower colocalization in the treatment but with larger differential mean colocalization (>0.07) and high significance (p-value ≤ 0.0001, Wilcoxon rank sum), were CD55 (DAF), CD44, CD18, CD11a, CD47, CD197 (CCR7), and CD84. DAF regulates the complement system on the cell surface that impairs the formation of the membrane attack complex (MAC), and another protein, CD59, is the MAC-inhibitory protein. CD59 scored higher in colocalization with CD20 and CD82 than CD55, but the mean difference against the control experiment was smaller.

Finally, we calculated the colocalization of order 4 for CD20, CD82, and CD37 with all other non-control proteins (**Supplementary Figure 14**). Unexpectedly, the three proteins (i.e., CD82, CD54, and CD37) obtaining the highest pairwise colocalization with CD20 (**Figure 4B**), not counting in the major histocompatibility proteins, were colocalized with high scores in both the control and treatment (>0.20), thus achieving lower significance (0.001< p-value ≤ 0.01, Wilcoxon rank test) (**Supplementary Figure 14**). Any of CD86, HLA-ABC, or HLA-DR that were high pairwise scoring with CD20 failed to achieve any significance (p-value > 0.01, Wilcoxon rank test) with CD20, CD82, and CD37 when compared to the control experiment (**Supplementary Figure 14**).

### Abundance and colocalization provide different biological aspects

**Figure 6:**
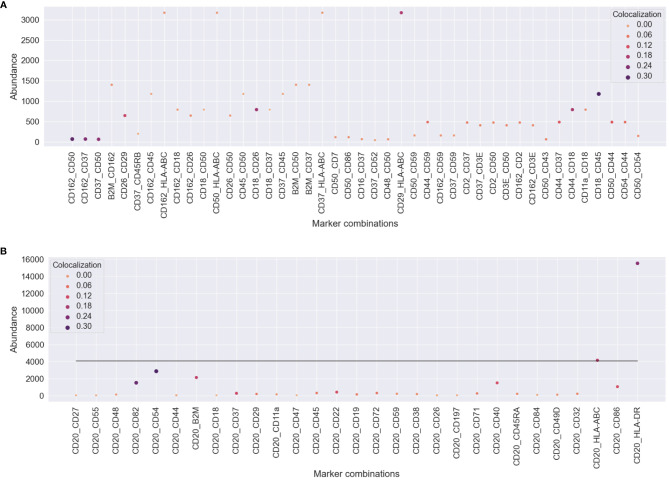
Comparison of pairwise colocalization and abundance on the proteins of interest for both experiments. **(A)** In the uropod stimulation experiment, the abundance is given by the maximum number of counts of the two compared proteins (y-axis), and colocalization is given as Spearman’s correlation (size and color). Some protein pairs indicate that high colocalization may be found occasionally when one of the proteins is highly abundant, e.g., CD29 and HLA-ABC, but not always, e.g., CD37 and HLA-ABC. **(B)** For the RTX-treated sample, all comparisons are made between CD20 and the proteins of interest. The abundance axis reflects the counts of the proteins of interest, and the line shows the averaged abundance of CD20 across all treated cells. RTX, rituximab.

To understand how protein abundance and colocalization measure different aspects of cellular responses to the environment and stimuli, we compared pairwise colocalization and protein abundance as raw molecule counts. We chose different pairs of proteins of interest in both experiments and plotted both the most abundant protein of the pair and the pairwise colocalization averaged across cells. **Figure 6** shows no inflation in our pairwise colocalization measurements by abundance.

In the uropod experiment (**Figure 6A**), all the pairs that we found to be highly colocalized (CD37, CD162, CD50, CD44, and CD54) were not among the highly or mid-abundant proteins. Another highly scoring pair in colocalization, CD29 and HLA-ABC, is also highly colocalized due to the abundance of one of them. However, CD37, CD50, and CD162 colocalization with HLA-ABC was not influenced by its abundance, as they were confined to the bulge of the uropod.

In the RTX experiment (**Figure 6B**), CD20 was, on average, the third most abundant protein in the Raji cells after HLA-DR and HLA-ABC. Therefore, it was difficult not to perceive dependence on abundance, as CD20 pairwise colocalization was high with those two proteins (>0.10). The other two most colocalized proteins, CD54 and CD82, were the third and fifth most abundant, respectively. Also, B2M, CD40, and CD86 were some of the most abundant proteins with high colocalization to CD20.

## Discussion

We analyzed two publicly available MPX experiments with our adjusted local assortativity algorithm for the detection of polarized and colocalized proteins on the surface of single cells.

Cells that were stimulated to form uropods after fixation of PHA-stimulated blasts and treated with RANTES (CCL5) showed a high colocalization score in pairwise comparison of proteins associated with the uropod (CD50, CD162, and CD37) (16). Notably, a member of the tetraspanin family, CD37, has been described as playing a role in the cytoskeleton remodeling of actin filaments but has never colocalized with other uropod proteins such as CD50 or CD162 (21). CD37 is necessary for leukocytes to follow a CXCL1 chemotactic gradient as tested in CD37-deficient mice (39).

On the attachment side of the stimulated cells, αLβ2 integrin (CD11a/CD18 or LFA-1) pairwise colocalization is not as significant as expected. This is mostly due to the low abundance of CD11a, which is often at the threshold level of control isotypes and leads, therefore, to generally lower scores in the stimulated cells. It is possible that the experimental conditions by fixing CD54 coating and posterior cleavage by enzymatic reaction may have affected the protein complex structure as well as epitope availability of CD11a.

Intercellular adhesion molecules (ICAMs) are arguably some of the best-annotated proteins in migrating immune cells (16). At a higher order of magnitude, we found that ICAMs scored much more significantly at order 3 and beyond. However, our colocalization method was able to distinguish that ICAM1–3 (CD54, CD102, and CD50) together at order 3 were not highly significant compared to the control. It is possible that ICAMs selectively group together and become more structurally significant in a larger cell membrane area that punctuates the colocalization of other proteins in pairs. Direct colocalization of CD18 with ICAM1 or with ICAM3 in *trans-*interactions has been reported through microscopy (40), but we cannot discard that *cis-*interactions may occur in our migratory model system. It has been observed that β2 integrin bending on human neutrophils rolling on a microfluidic device coupled to advanced microscopy facilitates interaction with ICAMs in *cis-*, thus inhibiting leukocyte adhesion *in vitro* and *in vivo* (41). On that system, they are able to prove that ICAM3 is the dominant LFA-1 ligand in *cis-* and that inhibition of the interaction between Mac-1 (C11b/CD18) and ICAM1 in *cis-* limits significantly neutrophil accumulation.

Pairwise colocalization signals on CD26 and CD29 have been reported in healthy mouse myofibroblasts in the past (42). Being present in most cell types, CD26 plays a double functionality as an immune-regulatory and proteolytic enzyme. CD26 can be found integral to both the membrane and its soluble form (43). This multifunctional protein is able to influence T-cell proliferation and chemotaxis but also truncate RANTES and alter the sub-receptor specificity of the cleaved chemokine (44). CD26 has a key role in adhesion and invasion for several cancer cells and has therefore become an established cell surface marker in serum (45). The extracellular matrix (ECM) is able to provide cells with co-stimulatory signals through different receptor–ligand interactions. Collagen has been described to provide proliferation signals to CD4+ cells via the CD3 pathway with the mediation of VLA-3 (CD49c/CD29) and CD26 receptors (46). Different adhesion factors of the very late activation antigen (VLA) family, sharing a common β1 subunit (CD29 or ITGB1), are able to receive signals either directly or indirectly to different proteins of the ECM and CD26 to collagen type I, IV, and fibronectin (47). Furthermore, on the pairwise colocalization effect of CD29 with HLA-ABC, certain isotypes of HLA-B are able to decrease ITGB1 expression and affect pancreatic cancer cell migration with contrasting effects (48).

CD44 is a transmembrane glycoprotein presenting ubiquitous expression and is able to bind to several ECM proteins (49). Some sources suggest that CD44 and CD18 may colocalize to mediate lymphocyte rolling and adhesion (50) and that CD44 interacts with the β2 subunit (CD18) of the LFA-1 integrin in lymphocytes (51) (52) and in colon cancer cells (53). CD44 is known to be expressed on cancer stem cells and implicated in many cancers as a marker of tumor burden and metastatic potential due to its numerous variant isoforms (49). Also, CD44 is a signaling partner in relation to cell growth, survival, and differentiation (54). As a therapeutic target, CD44 has held some promise in the past, e.g., anti-CD44 mAb therapy in breast cancer xenografts, reducing tumor growth and relapse post-chemotherapy (55). Despite recent disappointments in late-phase trials (56), still, new avenues are explored, e.g., nanoparticles (57) or carbon nanotubes (58), and hope remains on CD44 as a target as well as on better stratification of the patient population (56).

RTX is one of the pioneer biological therapies effective in many B-cell malignancies, such as chronic lymphocytic leukemias, non-Hodgkin’s, and Burkitt’s lymphomas. The human IgG1 Fc portion of RTX is capable of activating several mechanisms to cause cell death: complement-dependent cytotoxicity (CDC), complement-dependent cellular cytotoxicity, antibody-dependent cellular phagocytosis, or antibody-dependent cellular cytotoxicity (59). The relative killing efficiencies of RTX have been well studied *in vitro*, but the *in vivo* precise mechanism of action remains elusive (60), and better understanding is still needed to impede disease relapsing. In order to design for improved effects, different IgG subtypes have been engineered and studied both *in vitro* with Ramos cells (61) and lymphoma B-cell organoids (62).

Our analysis shows that, upon RTX treatment, CD55 and CD59 are colocalized with CD20 via the CD82 tetraspanin, whereas the direct pairwise colocalization with CD20 of both proteins was not significantly differentiated from controls. It suggests that CD55 and CD59 are indirectly associated with CD20 via a tetraspanin network, resembling the CD46 association with many β1 integrins and tetraspanins (63). This may indicate that targeting inhibitors of CDC may achieve superior killing, as it has been suggested by others (64).

Our data also support that CD82, but not CD9, colocalizes with CD19 and CD20 (65). Unfortunately, at the time of writing, some important proteins that play a crucial part in the CD20 therapeutic “enigma” (59) are not present on the current MPX AOC panel, among them, CD46, another complement inhibitory component; CD21 (CR2), the complement C3d receptor; and CD81 (TAPA-1), another tetraspanin. The trio of proteins, CD21, CD19, and CD81, form the CR2–CD19–CD81 complex, often called the B-cell co-receptor complex that enhances BCR signaling (66).

We also found another tetraspanin, CD37, suggested to be part of a multicomponent supramolecular complex, so-called “tetraspans-DR complexes”. After solubilization of membranes of human B-cell lines and tonsillar B cells, seven components were discovered by co-precipitation together with HLA-DR antigens: four of the tetraspanins present in B cells (CD37, CD53, TAPA-1, and R2/C33), as well as CD19 and CD21 (67). The same laboratory employed later another technique, flow cytometric energy transfer, to find three tetraspan molecules (CD53, CD81, and CD82) complexed with MHC class I, MHC class II, and CD20 on the surface of a human B-cell line (37). Recently, CD20 and CD37 have been confirmed to form a complex by a proximity ligation assay (68). In this preprint, it is hypothesized that the presence of CD20 stabilizes CD37 in the cell membrane as increased internalization of anti-CD37 is measured on deficient CD20 lymphoma B-cell lines (68).

The potential of CD37 as a therapeutic target has been recognized by developing biparatopic antibodies with engineered Fc chains that form IgG hexamers (69) and, in clinical trials (NCT01317901), exploring combinatorial therapies for relapsed patients and good overall response rate (70). Bobrowicz and colleagues recently tested that upon diminished levels of CD37 in different cell lines, even with downregulation of CD20, cytotoxicity of CAR-T cells was not significantly impaired. Therefore, in their opinion, CD37 remains an attractive therapeutic target (68).

Overall, we want to highlight the complexity and dynamism of the cellular membrane driven by tetraspanins, integrins, and adhesion molecules. We find several molecules in common to both datasets that colocalize together upon very different stimuli. Tetraspanin-enriched microdomains facilitate the compartmentalization of specialized receptors and adhesion molecules in membrane domains that connect to the underlying intracellular architecture of the cell (71, 72).

One of the main caveats of colocalization analysis is the difference between the abundance and true signal. CD20 is the third molecule in mean abundance in the RTX experiment and presents high pairwise colocalization with, e.g., HLA-DR. These macro-complexes have been well described in the literature (37), but highly abundant proteins may colocalize with all other proteins by chance. In the uropod experiment, the highly colocalizing pairs have low mean abundances compared to the highly abundant proteins distributed uniformly across the area of the cell, e.g., HLA-ABC, whereas the CD20 cap on a Raji cell after RTX treatment is likely a much larger area than the smaller and well-constrained uropod bulge and, also, more prone to accidental overlap by low- and high-abundance proteins. While these are two very different cellular responses and biological systems, the area of polarization and overlap may warrant different interpretations.

Even though the local assortativity is improving on this by taking the spatial aspect of the graph into account, there is still some bias toward abundant proteins on the cell surface. Although the interesting signal in our method is likely going to be indicated by low abundant proteins showing high colocalization, inflation of colocalization scores at higher orders of comparison has been observed, and strategies to ameliorate this may use corrections from lower orders of comparison (i.e., correct scores on order 3 with scores from order 2). We think that the major confounding factor to colocalization measurements is abundant proteins. Improvements to our method in this area may consider the idea of richness of species when comparing values across sites (73). However, we have shown how the abundance and colocalization of proteins measure different aspects of cell biology, both equally important.

MPX capabilities in throughput, sensitivity, and three-dimensional field of view (14) create exceptional conditions to study protein constellations at single-cell resolution. Here, we expand on the concept of local assortativity as presented in the Peel et al. study (15) for labeled nodes in networks to capture the influence of both the structure of the cell represented in the graph and the location distribution of each protein. Adjusted local assortativity provides a parameter-free algorithm that calculates the colocalization of molecules, avoiding the complex problem of finding neighborhoods of clustered features by different approaches (74, 75).

Local assortativity could be used with other single-cell technologies and experimental designs. However, it is required for it to work in that data were processed in the form of networks with labels and features in the vertices. With the MPX technology, cells and their protein molecules are modeled in the form of graphs. With this method, we improved the global scores provided in the Karlsson et al. study in terms of polarization and colocalization to find continuous and bounded measures of the biological phenomena assayed by MPX. Furthermore, we built on the pairwise local assortativity using a multi-site similarity method used from an ecological context (32) in order to achieve multiple protein comparisons from groups of more than two proteins. With pairwise and multi-way comparison, we aimed to capture detailed structural properties of the cell graphs and facilitate the comparison of molecules colocalizing among groups of proteins in a more detailed way. The scoring methods devised for pairwise and higher-order colocalization are different, so a direct comparison of score levels across those two is not possible at the moment. The higher order of colocalization is very useful for hypothesis testing, but we foresee its use rather as a tool for specific *in silico* experiments guided by prior knowledge.

MPX with pairwise and a higher order of colocalization yields deep phenotyping not achievable with other assays by measuring 76 proteins (and four controls) at the same time in a single experimental workflow. This is a throughput of several orders of magnitude higher in plexity at a reduced experimental time from sample to processed data over what can be achieved with confocal and super-resolution microscopy. The experiments presented in this report took approximately 2 weeks to complete. However, given the exponential number of protein combinations, there is a clear need to develop algorithms and tools to exploit this novel data type.

Another advantage of MPX data is that they achieve single-cell resolution in one experiment of 300–1,000 cells to study variability in response to stimuli. We have measured effect sizes against controlled experiments but have not explored yet the complexity of responses in terms of dividing treatment and controls in different subgroups. We should also consider the combinatorial multiplexity of single-cell studies, and the comparison of experimental conditions is therefore a vital tool in the analysis of colocalization, which begins with a good study design.

Our findings underpinned by MPX together with our novel computational method may provide avenues for hypothesis-driven therapeutic design that explores spatially colocalized protein constellations in the cell.

## Data availability statement

Pixelator output data from MPX libraries are made publicly available under a Creative Commons Share Alike License at https://software.pixelgen.com/datasets. Accompanying analysis and data for the purpose of this paper is released under GPL-2 license at https://github.com/PixelgenTechnologies/adjusted-local-assortativity-paper. Pixelator is available under MIT license at https://github.com/PixelgenTechnologies/pixelator and the pipeline orchestrator is released as a nf-core workflow at https://github.com/nf-core/pixelator under MIT license.

## Ethics statement

Only publicly available MPX datasets were used for this study. The microscopy pictures shown in **Figure 1** were obtained in the Karlsson et al. (14) study, where all the ethics statements can be found and cell lines providers are listed.

## Author contributions

JR: Conceptualization, Data curation, Formal analysis, Investigation, Methodology, Software, Visualization, Writing – original draft, Writing – review & editing. MK: Writing – review & editing. DT: Writing – review & editing. TK: Writing – review & editing. FK: Writing – review & editing. SF: Writing – review & editing. JD: Conceptualization, Writing – review & editing. AB: Conceptualization, Data curation, Funding acquisition, Investigation, Methodology, Project administration, Resources, Software, Supervision, Writing – original draft, Writing – review & editing.

## Footnotes

1
https://software.pixelgen.com/datasets/.

2
https://software.pixelgen.com/datasets/uropod-t-cells-v1.0-immunology-I.

3
https://www.atcc.org/products/ccl-86.

4
https://software.pixelgen.com/datasets/cd20-rituximab-v1.0-immunology-I.
