# Supplementary material for: Using adjusted local assortativity with Molecular Pixelation unveils colocalization of membrane proteins with immunological significance
Source: Front Immunol. 2024 Jun 25;15:1309916. doi: 10.3389/fimmu.2024.1309916 (PMC11231075; doi:10.3389/fimmu.2024.1309916)
Supplement: Supplementary file 1 [file DataSheet_1.pdf]

Supplementary Material

1 Supplementary Data

1.1 Optimizing the PageRank threshold.

In order to optimize the runtime compared to the original implementation by Peel *et al.*, we try to adjust the PageRank threshold. The personalized PageRank vector is calculated by approximating an integral over the different distributions of local neighborhoods. These distributions are calculated from 0 to 1 with 0.1 steps, where 0 is direct neighborhood and 1 traverses the graph, as explained in Peel *et al*. This approximation has a threshold of 1e-9 in the original paper. We are interested in finding the optimum as by increasing the threshold we might improve runtime at similar accuracy. A clear drop off in difference between the original values and the selected threshold occurs at 1e-4 (Supplementary Figure 1) that becomes our local assortativity threshold. The mean difference is an absolute value and on a per node basis it would equal to a mean difference of less than 0.002 in local assortativity score at the node whereas the overall computational time is drastically reduced.

1.2 Proof upper and lower bound adjusted local assortativity

Let $G = (V, E)$ be a graph as described in the “Molecular Pixelation” methods section. Then V is a set of vertices $V = \{v_1, v_2, ..., v_n\} \neq \emptyset$. We can now calculate for each vertex v_i the local assortativity x_i and perform the transformation as described in the method section “Bound version of the local assortativity”. Then we have

$$\varrho: \mathbb{R} \to \mathbb{R}, \; x_j \to z_j \text{ with } \varrho(x_j) = (h \circ g \circ f)(x_j).$$

Then we have for f as described before

$$f(x_j) = \frac{x_j}{\sum_{i=1, x_i > 0}^{n} x_i} \text{ for } x_j \geq 0 \text{ and } \sum_{i=1}^{n} |x_i| > 0,$$

$$f(x_j) = \frac{x_j}{\sum_{i=1, x_i < 0}^{n} |x_i|} \text{ for } x_j < 0 \text{ and } \sum_{i=1}^{n} |x_i| > 0$$

$$\text{and } f(x_j) = 0 \text{ for and } \sum_{i=1}^{n} |x_i| = 0.$$

This leaves $\tilde{x}_j = f(x_j)$ bound from minus one to one. Using this we can rewrite $\varrho(x_j)$ using the bound variable $\tilde{x}_j$ and get

$$\varrho(x_j) = (h \circ g \circ f)(x_j) = (h \circ g)(\tilde{x}_j) = \log\left(\frac{\tilde{x}_j}{\sigma} + 1\right)$$

By filling in the standard deviation σ and knowing that the transformation f has set the mean $\mu = 0$ we get

$$\log\left(\frac{\tilde{x}_j}{\sigma} + 1\right) = \log\left(\frac{\tilde{x}_j}{\sqrt{\frac{1}{n}\sum_{i=1}^{n}\left(\tilde{x}_i - \mu\right)^2}} + 1\right) = \log\left(\frac{\tilde{x}_j}{\sqrt{\frac{1}{n}\sum_{i=1}^{n}\left(\tilde{x}_i\right)^2}} + 1\right).$$

Case I; $X, Y \neq \emptyset$**:**

Under the preliminaries of the case we can assume that we have both positive and negative values for $\{\tilde{x}_1, \tilde{x}_2, \ldots, \tilde{x}_n\}$. By definition we know therefore we have two subset of $\{\tilde{x}_1, \tilde{x}_2, \ldots, \tilde{x}_n\}$, given by

$$X = \{r_1, r_2, \ldots, r_N\} \text{ and } Y = \{q_1, q_2, \ldots, q_M\} \text{ with } \sum_{i=1}^{N} r_i = 1 \text{ and } \sum_{i=1}^{M} q_i = -1.$$

Therefore we can split the standard deviation into two sums and get

$$\log\left(\frac{\tilde{x}_j}{\sqrt{\frac{1}{n}\sum_{i=1}^{n}\left(\tilde{x}_i\right)^2}} + 1\right) = \log\left(\frac{\tilde{x}_j}{\sqrt{\frac{1}{n}\left(\sum_{i=1}^{N} r_i^2 + \sum_{i=1}^{M} q_i^2\right)}} + 1\right).$$

As we know the sums of X and Y we can now use the Cauchy-Schwarz inequality for real numbers in both cases

$$\left(\sum_{i=1}^{N} r_i \cdot 1\right)^2 \leq \left(\sum_{i=1}^{N} r_i^2\right) \cdot \left(\sum_{i=1}^{N} 1^2\right) \Leftrightarrow \left(\sum_{i=1}^{N} r_i\right)^2 \leq \left(\sum_{i=1}^{N} r_i^2\right) \cdot N \Leftrightarrow \frac{1}{N}\left(\sum_{i=1}^{N} r_i\right)^2 \leq \sum_{i=1}^{N} r_i^2 \Leftrightarrow \frac{1}{N} \leq \sum_{i=1}^{N} r_i^2,$$

$$\left(\sum_{i=1}^{M} q_i \cdot 1\right)^2 \leq \left(\sum_{i=1}^{M} q_i^2\right) \cdot \left(\sum_{i=1}^{M} 1^2\right) \Leftrightarrow \left(\sum_{i=1}^{M} q_i\right)^2 \leq \left(\sum_{i=1}^{M} q_i^2\right) \cdot M \Leftrightarrow \frac{1}{M}\left(\sum_{i=1}^{M} q_i\right)^2 \leq \sum_{i=1}^{q} q_i^2 \Leftrightarrow \frac{1}{M} \leq \sum_{i=1}^{M} q_i^2.$$

As the square root function and the logarithm are both monotonically increasing for positive values we get

$$\log\left(\frac{\tilde{x}_j}{\sqrt{\frac{1}{n}\left(\sum_{i=1}^{N} r_i^{\,2} + \sum_{i=1}^{M} q_i^{\,2}\right)}} + 1\right) \le \log\left(\frac{\tilde{x}_j}{\sqrt{\frac{1}{n}\left(\frac{1}{N} + \frac{1}{M}\right)}} + 1\right) = \log\left(\frac{\sqrt{n}\cdot\tilde{x}_j}{\sqrt{\frac{1}{N} + \frac{1}{M}}} + 1\right).$$

Using again the monotony of the square root function and the logarithm for positive number we can make another estimation for an upper bound given by

$$\log\left(\frac{\sqrt{n}\cdot\tilde{x}_j}{\sqrt{\frac{1}{N} + \frac{1}{M}}} + 1\right) \le \log\left(\frac{\sqrt{n}\cdot\tilde{x}_j}{\sqrt{\frac{1}{\max\limits_{v\in\{N,M\}} v} + \frac{1}{\max\limits_{v\in\{N,M\}} v}}} + 1\right) = \log\left(\frac{\sqrt{n}\cdot\tilde{x}_j}{\sqrt{\frac{2}{\max\limits_{v\in\{N,M\}} v}}} + 1\right) \le \log\left(\frac{\sqrt{n}\cdot\tilde{x}_j}{\sqrt{\frac{2}{n}}} + 1\right)$$

$$= \log\left(\frac{\sqrt{n}\cdot\sqrt{n}\cdot\tilde{x}_j}{\sqrt{2}} + 1\right) = \log\left(\frac{n\cdot\tilde{x}_j}{\sqrt{2}} + 1\right).$$

Case II; $X \neq \emptyset$ **and** $Y = \emptyset$ **or** $X = \emptyset$ **and** $Y \neq \emptyset$ **:**

Assuming now that $X \neq \emptyset$ and $Y = \emptyset$ without loss of generality, than we get under the same assumptions as before

$$\log\left(\frac{\tilde{x}_j}{\sqrt{\frac{1}{n}\sum_{i=1}^{n}\left(\tilde{x}_i\right)^2}} + 1\right) = \log\left(\frac{\tilde{x}_j}{\sqrt{\frac{1}{n}\left(\sum_{i=1}^{N} r_i^{\,2}\right)}} + 1\right).$$

As we know the sum of X we can now use the Cauchy-Schwarz inequality for real numbers and this yields as before

$$\left(\sum_{i=1}^{N} r_i\cdot 1\right)^2 \le \left(\sum_{i=1}^{N} r_i^{\,2}\right)\cdot\left(\sum_{i=1}^{N} 1^2\right) \Leftrightarrow \frac{1}{N} \le \sum_{i=1}^{N} r_i^{\,2}.$$

As the square root function and the logarithm are both monotonically increasing for positive values we get

$$\log\left(\frac{\tilde{x}_j}{\sqrt{\frac{1}{n}\left(\sum_{i=1}^{N} r_i^{\,2}\right)}} + 1\right) \le \log\left(\frac{\tilde{x}_j}{\sqrt{\frac{1}{n}\left(\frac{1}{N}\right)}} + 1\right) = \log\left(\frac{\sqrt{n}\cdot\tilde{x}_j}{\sqrt{\frac{1}{N}}} + 1\right)$$

$$\Rightarrow \log\left(\frac{\sqrt{n}\cdot\tilde{x}_j}{\sqrt{\frac{1}{N}}} + 1\right) \le \log\left(\frac{\sqrt{n}\cdot\tilde{x}_j}{\sqrt{\frac{1}{n}}} + 1\right) = \log\left(n\cdot\tilde{x}_j + 1\right).$$

Case III; $X,\ Y = \emptyset$**:**

The case of both $X, Y = \emptyset$ cannot exist, as the graph we define as the input data for the transformation must at least contain one vertex and therefore at least one of the two sets must not be empty.

Therefore we get that the function $\varrho(x_j) = (h \circ g \circ f)(x_j)$ is bound, as the function is symmetrical with the center at zero, therefore we can also deduct the lower bound based on the same proof with negative signum. Let n now be, as before, the number of vertices, then this gives us the boundaries, of the function as

$$\left[-\log\left(n \cdot \tilde{x}_j + 1\right), \log\left(n \cdot \tilde{x}_j + 1\right)\right].$$

It has been proven that the normalized local assortativity $\tilde{x}_j$ is known to have a maximum of 1 and a minimum of -1, making the transformation symmetrical on both sides retaining the sign of the original values. Thus, we can substitute $\tilde{x}_j$ with 1 getting the final limits as

$$[-\log(n + 1), \log(n + 1)].$$

1.3 Permutation testing

To adjust the local assortativity for any potential count bias, a recalculated score for the same marker was derived, we simulated graphs with permuted marker count values (Supplementary Data 1.4). A null distribution representing the same cell graph with random marker localization was created by permutation and recalculation of local assortativity 75 times per cell. An adjusted score per marker was then calculated as the difference between the observed score and the null distribution mean (Supplementary Figure 4).

As seen in Supplementary Figure 4 it is hard to distinguish any visible change in the CD50 hotspot region with high adjusted local assortativity scores. With randomization of antibody labels and counts in homogeneously connected cell graphs, the structural effects become non-significant producing scores close to zero but not exactly zero (*i.e.* zero means uniform mixing). As permutation produces a number close to zero (Supplementary Figure 5), when applying it to adjust local assortativity, it would have a very small adjustment factor at the expense of significantly increasing runtime from seconds per cell to minutes. Thus, the total runtime per sample with permutations and calculation of adjusted local assortativity would increment from a couple of hours to days per sample. The relatively small changes to the graph from the shuffling can be seen in Supplementary Figure 5, where the non shuffled values display a strong polarization for CD50 for the selected uropod cell, while the shuffled scores are mostly in the near zero range. This further highlights the point that the structure of the cell graphs is relatively homogenous and therefore displays little to no significant bias on the scores based on the graphs structure. Therefore we skip the permutation process to save runtime on a full experiment as the adjustment factor is very small.

The permutation testing feature, however, as implemented in the code[1] from Peel *et al.* is kept (Data Availability Statement) as it might prove useful for smaller datasets with high variance in structure.

1.4 Finding the optimal number of permutations to correct for abundance

As the local assortativity is influenced by the underlying graph structure Peel *et al.*, we try to reduce this effect by using permutation testing. For this purpose, we need to select a number of permutations so the mean difference between the original local assortativity scores and the permuted local assortativity in a subset of components would approach a stable difference.

The threshold selection here is not as clear in this case as for the PageRank threshold but with an increase on permutations, the execution runtime of the algorithm would increment prohibitively. In Supplementary Figure 6, we show that the mean average score begins to stabilize at 75 permutations and thus we selected it as a threshold for local assortativity permutations.

[1] https://github.com/piratepeel/MultiscaleMixing

2 Supplementary Figures and Tables

2.1 Supplementary Figures

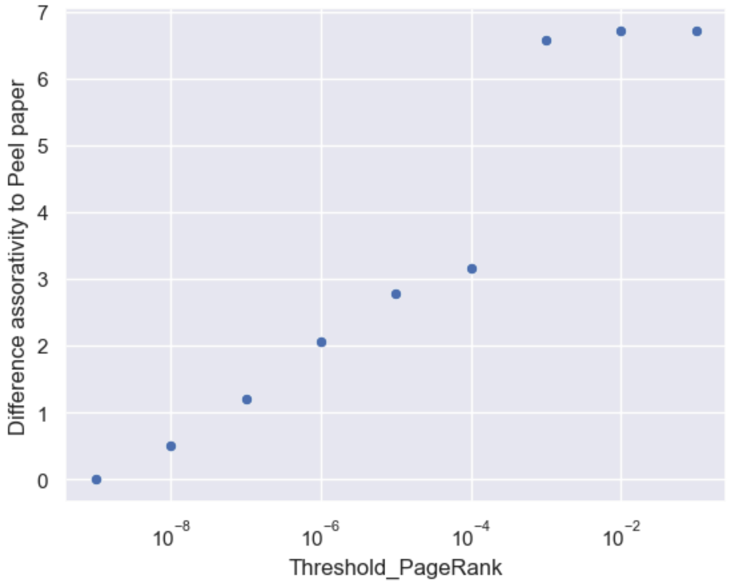

Supplementary Figure 1. The mean difference between the adjusted local assortativity scores with a threshold set as in Peel *et al.* and the adjusted local assortativity scores with different values for the PageRank threshold on 250 cell components subsampled.

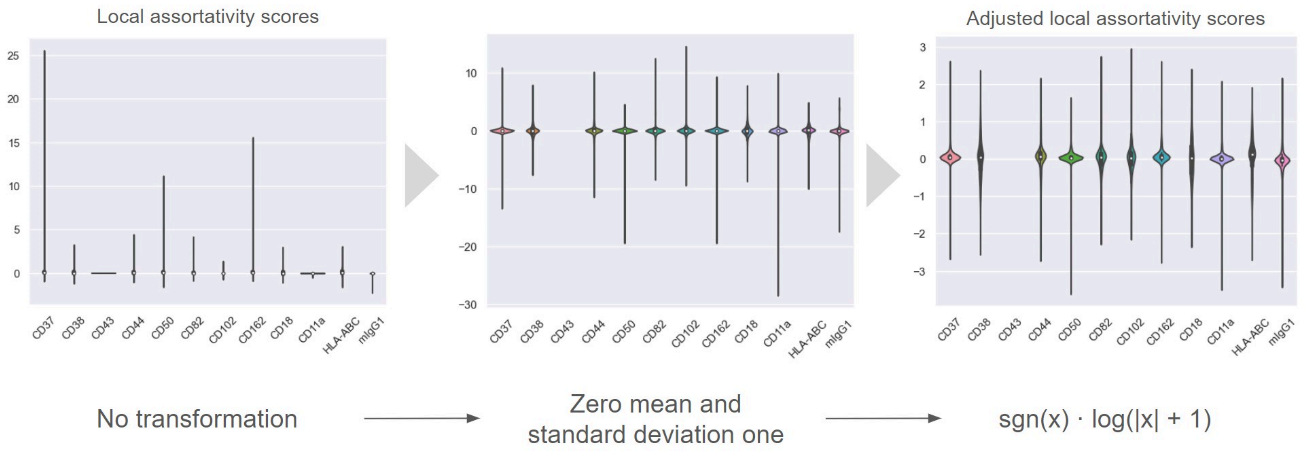

Supplementary Figure 2. Different steps are taken to transform local assortativity scores to adjusted local assortativity. First the mean is moved to zero while preserving the average distribution on both

sides, followed by setting the standard deviation to one and scaling the data with a log transformation.

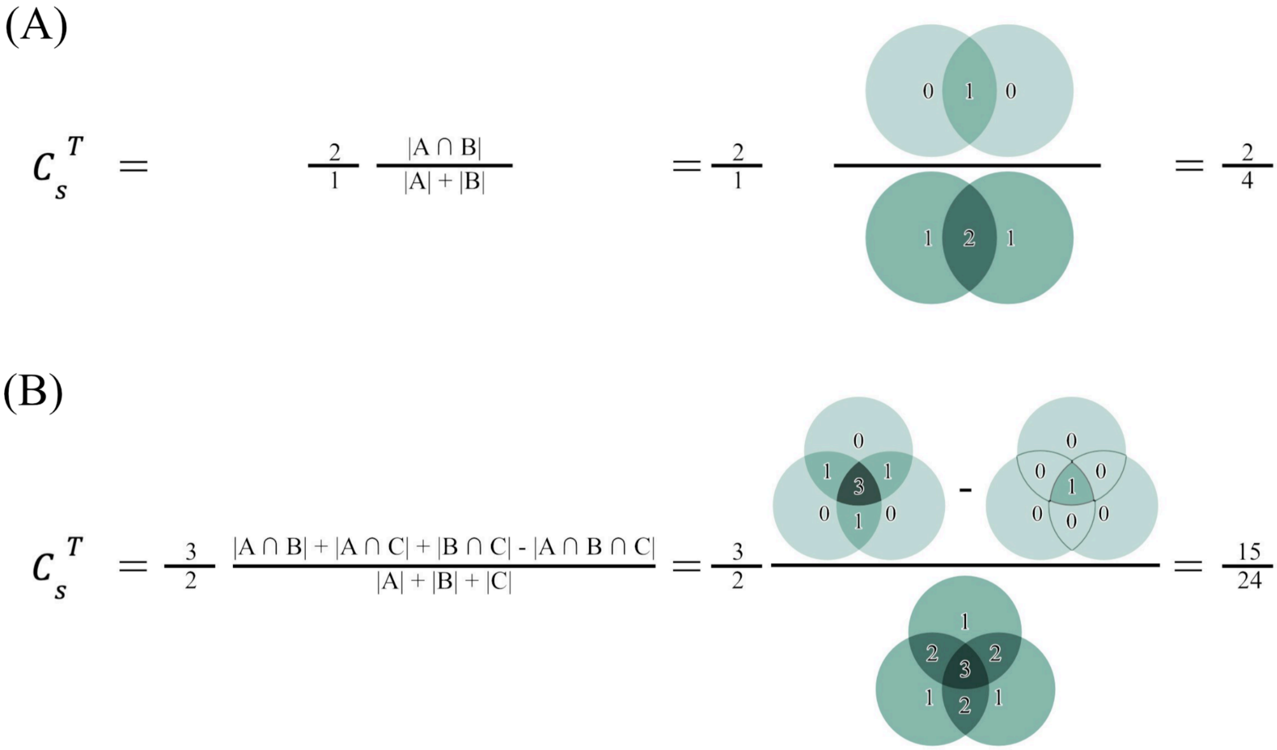

Supplementary Figure 3. Explantation of higher order comparisons with sets. Panel (A) shows the formula for the case T=2. In this case the multi-site similarity measure is equivalent to the Sørensen-Index. Panel (B) shows the case of T=3 and introduces the changing sign and illustrates the summation for multiple intersections.

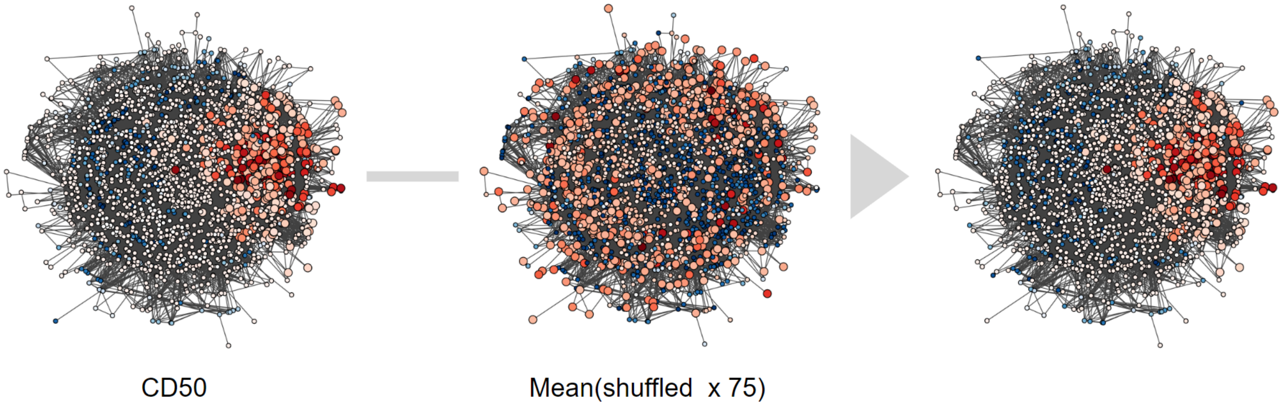

Supplementary Figure 4. The adjusted local assortativity for CD50 and the mean adjusted local assortativity for the shuffled protein values show a small effect on the highly polarized areas from the underlying graph structure.

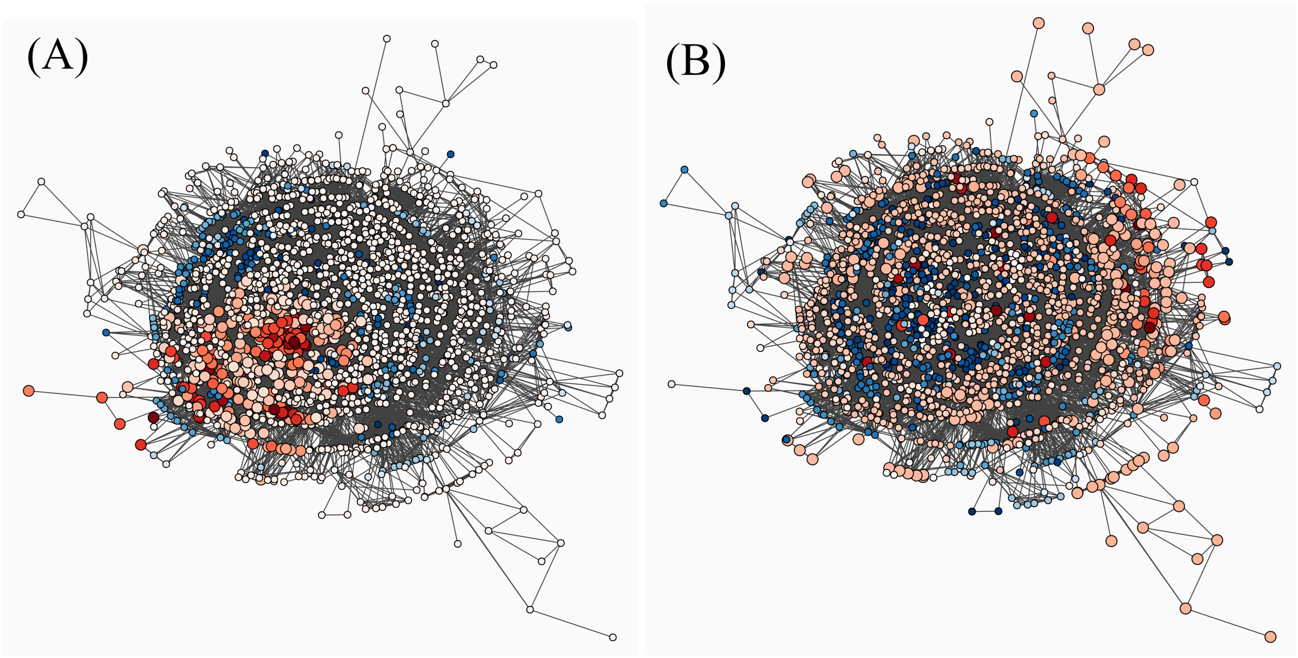

Supplementary Figure 5. Adjusted local assortativity scores for CD50, from the stimulated uropod sample, in the same cell when protein counts have been (A) non-permuted and (B) permuted. Abundant proteins do not hold a significant influence on the data when averaged over many permutations, indicating that local assortativity gives true signal.

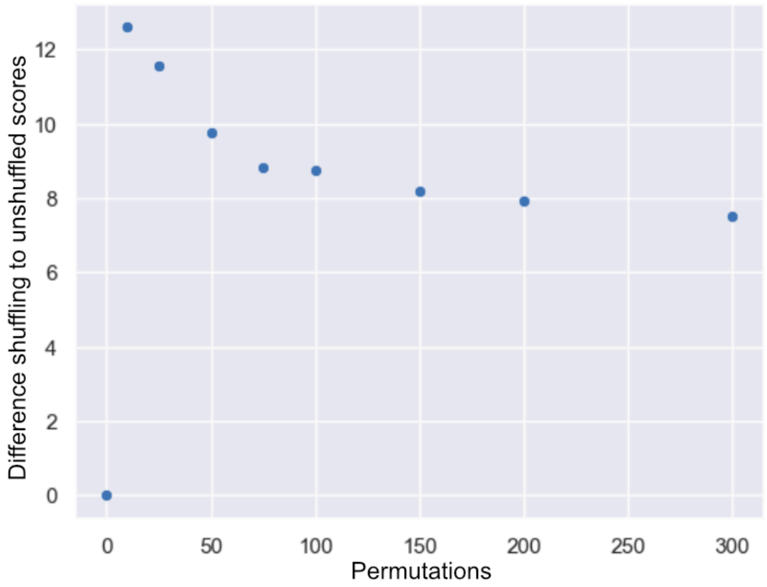

Supplementary Figure 6. The difference between local assortativity scores for multiple components across different numbers of permutations. For each certain number of permutations the same 50 random cell components were analyzed and compared to the original by a mean difference.

Supplementary Material

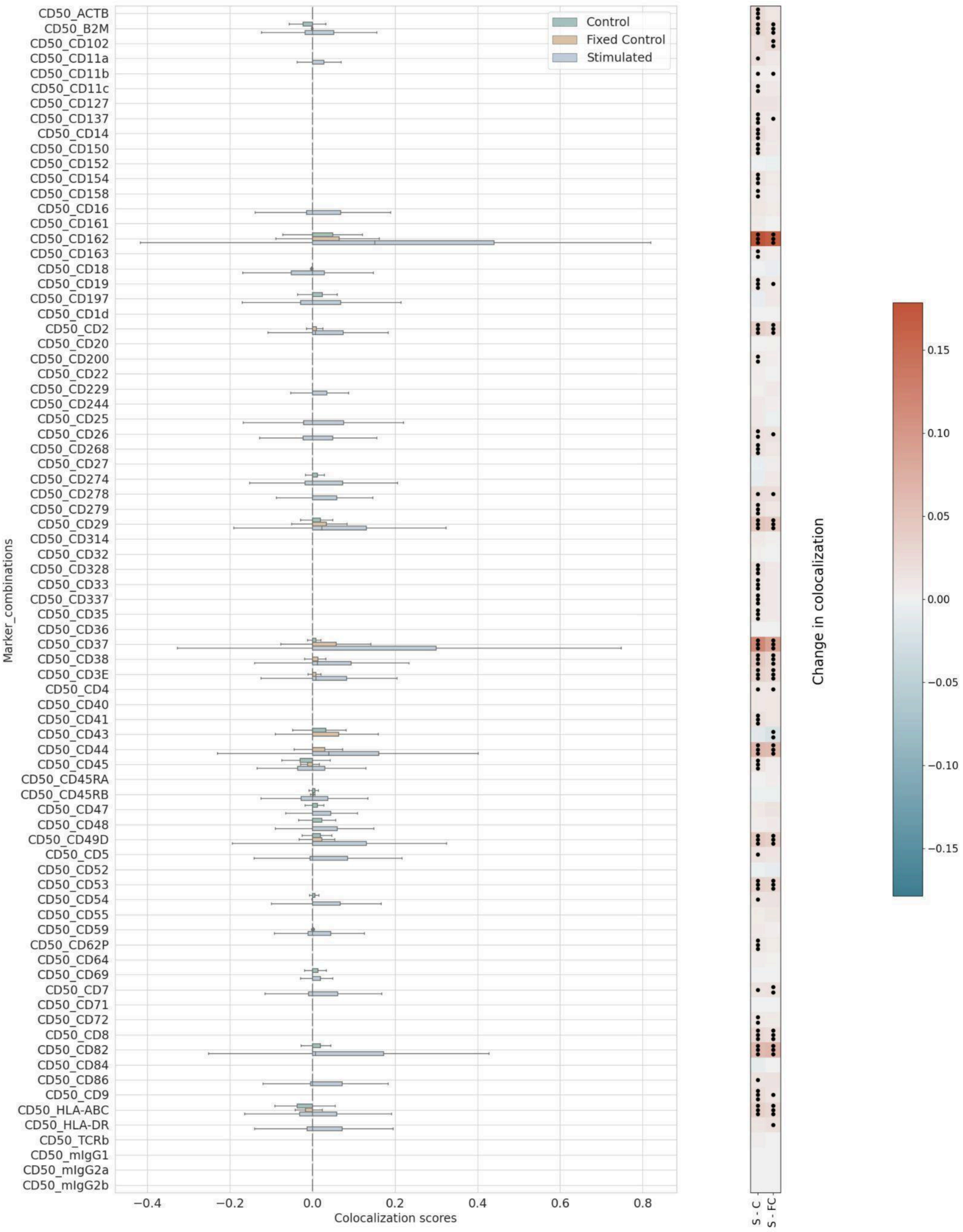

Supplementary Figure 7. Pairwise differential colocalization for CD50, from the uropod experiment, with all other proteins of the MPX antibody panel, including controls. The first column shows the score distribution across the three conditions. The last column displays the differential colocalization score when comparing stimulated to both control conditions and their significance. The dots indicate p-value ranges generated by a Wilcoxon rank test: 0 dots (p-value > 0.01), 1 dot (0.001 < p-value <= 0.01), 2 dots (0.0001 < p-value <= 0.001) and 3 dots (p-value <= 0.0001).

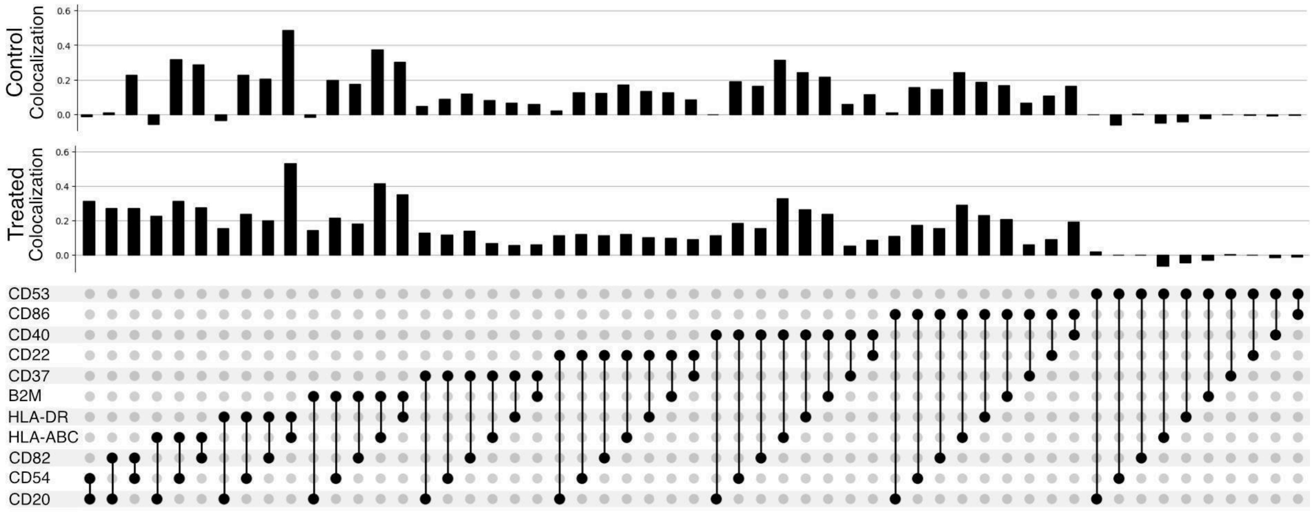

Supplementary Figure 8. Higher order colocalization of protein pairs in the Rituximab experiment, for all combinations of the proteins of interest. The first bar plot displays the higher order colocalization of pairs of proteins in the control sample. The second one shows the higher order colocalization scores in the fixed control sample and the last row shows the higher order colocalization scores in the simulated sample. The combinations of pairs of proteins compared are shown below with linked dots.

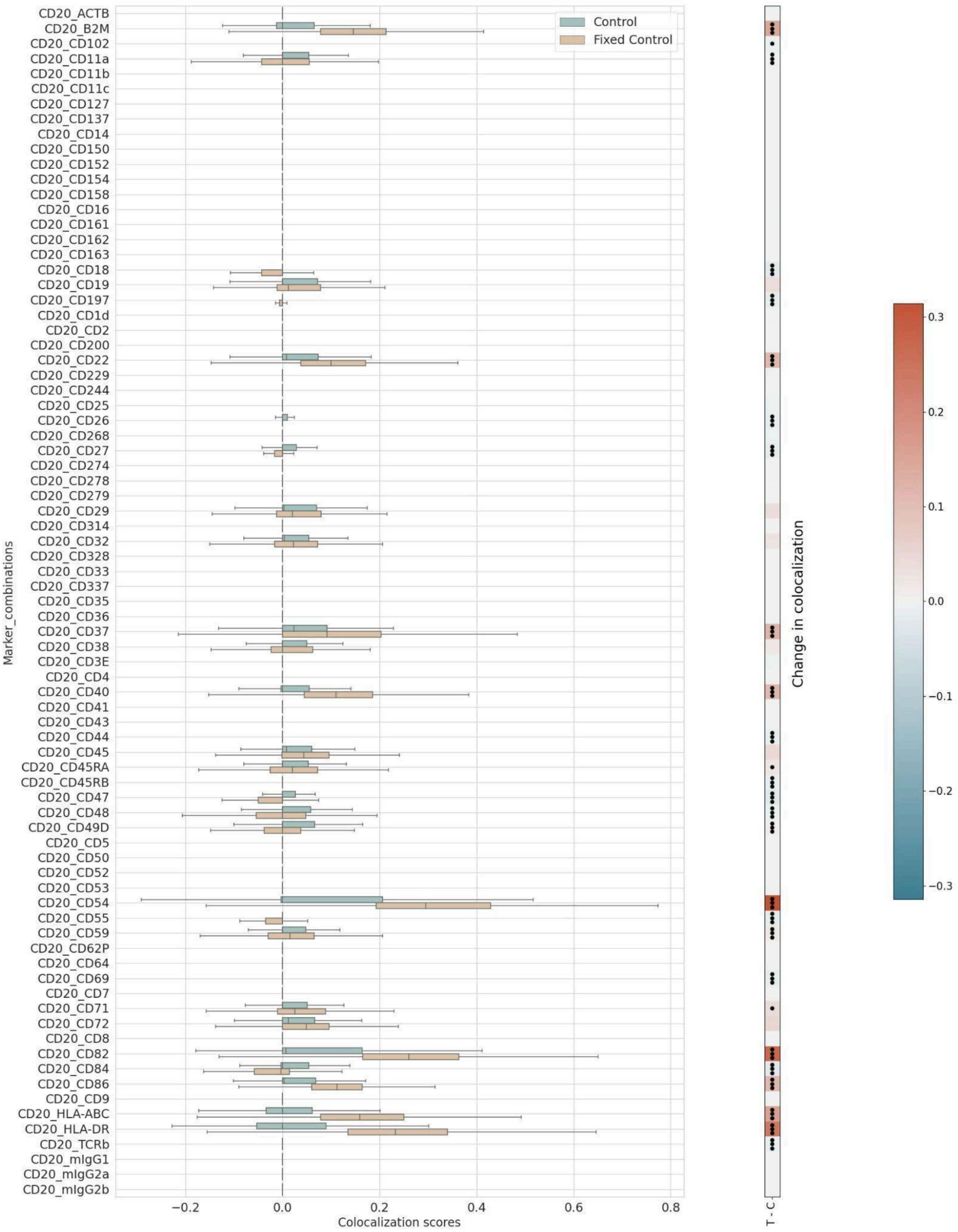

Control
Fixed Control
Marker_combinations
Colocalization scores
Change in colocalization
T - C
CD20_ACTB
CD20_B2M
CD20_CD102
CD20_CD11a
CD20_CD11b
CD20_CD11c
CD20_CD127
CD20_CD137
CD20_CD14
CD20_CD150
CD20_CD152
CD20_CD154
CD20_CD158
CD20_CD16
CD20_CD161
CD20_CD162
CD20_CD163
CD20_CD18
CD20_CD19
CD20_CD197
CD20_CD1d
CD20_CD2
CD20_CD200
CD20_CD22
CD20_CD229
CD20_CD244
CD20_CD25
CD20_CD26
CD20_CD268
CD20_CD27
CD20_CD274
CD20_CD278
CD20_CD279
CD20_CD29
CD20_CD314
CD20_CD32
CD20_CD328
CD20_CD33
CD20_CD337
CD20_CD35
CD20_CD36
CD20_CD37
CD20_CD38
CD20_CD3E
CD20_CD4
CD20_CD40
CD20_CD41
CD20_CD43
CD20_CD44
CD20_CD45
CD20_CD45RA
CD20_CD45RB
CD20_CD47
CD20_CD48
CD20_CD49D
CD20_CD5
CD20_CD50
CD20_CD52
CD20_CD53
CD20_CD54
CD20_CD55
CD20_CD59
CD20_CD62P
CD20_CD64
CD20_CD69
CD20_CD7
CD20_CD71
CD20_CD72
CD20_CD8
CD20_CD82
CD20_CD84
CD20_CD86
CD20_CD9
CD20_HLA-ABC
CD20_HLA-DR
CD20_TCRb
CD20_mIgG1
CD20_mIgG2a
CD20_mIgG2b
−0.2
0.0
0.2
0.4
0.6
0.8
0.3
0.2
0.1
0.0
−0.1
−0.2
−0.3

Supplementary Figure 9. Pairwise differential colocalization, from the Rituximab experiment, for CD20 with all other proteins of the MPX antibody panel, including controls. The first column shows the score distribution across the two conditions. The last column displays the differential colocalization score when comparing the Rituximab-stimulated cells to the control condition and their significance. The dots indicate p-value ranges generated by a Wilcoxon rank test: 0 dots (p-value > 0.01), 1 dot (0.001 < p-value <= 0.01), 2 dots (0.0001 < p-value <= 0.001) and 3 dots (p-value <= 0.0001).

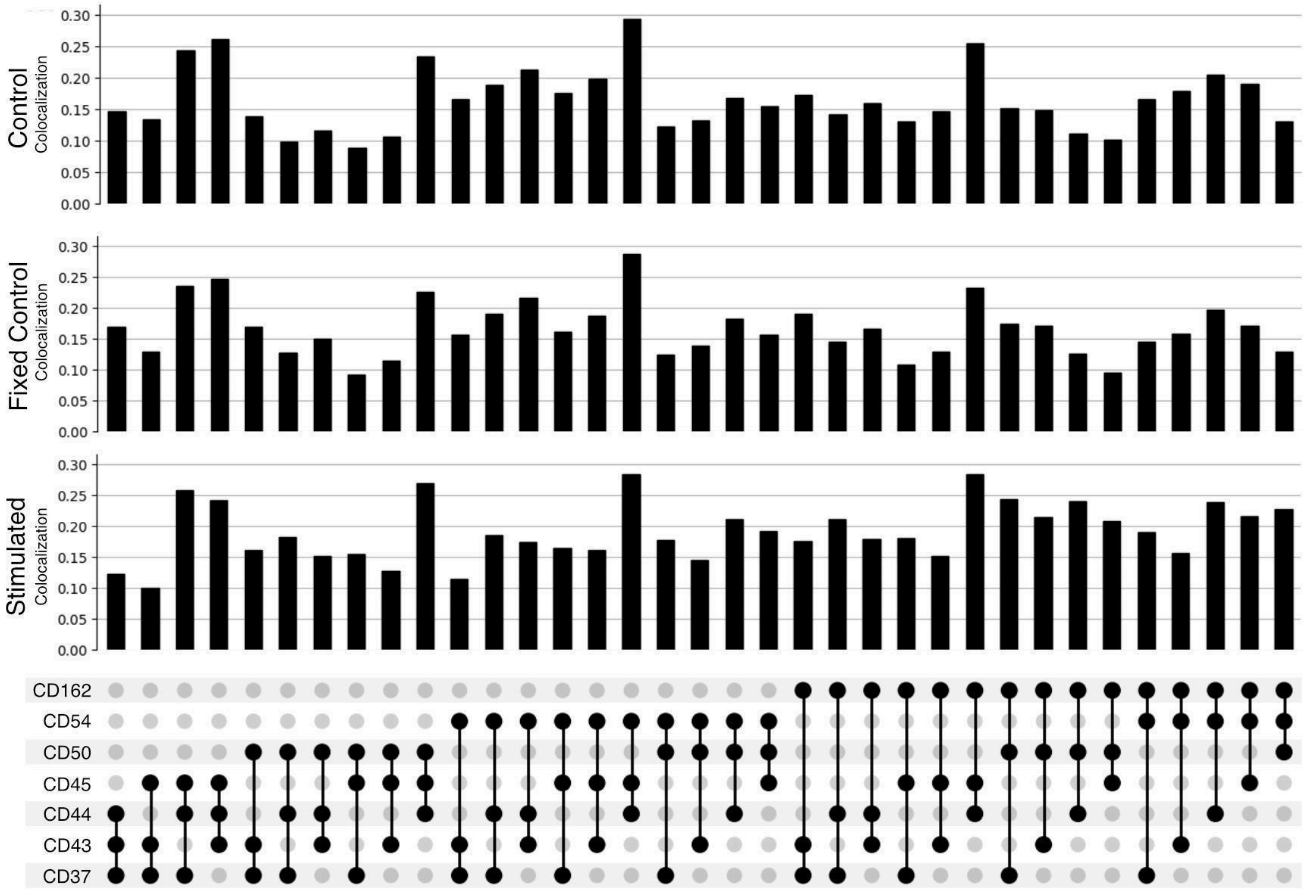

Supplementary Figure 10. Higher order colocalization of protein trios in the uropod experiment, for all combinations of the proteins of interest. The first bar plot displays the higher order colocalization of trios of proteins in the control sample. The second one shows the higher order colocalization scores in the fixed control sample and the last row shows the higher order colocalization scores in the simulated sample. The combinations of trios of proteins compared are shown below with linked dots.

Supplementary Material

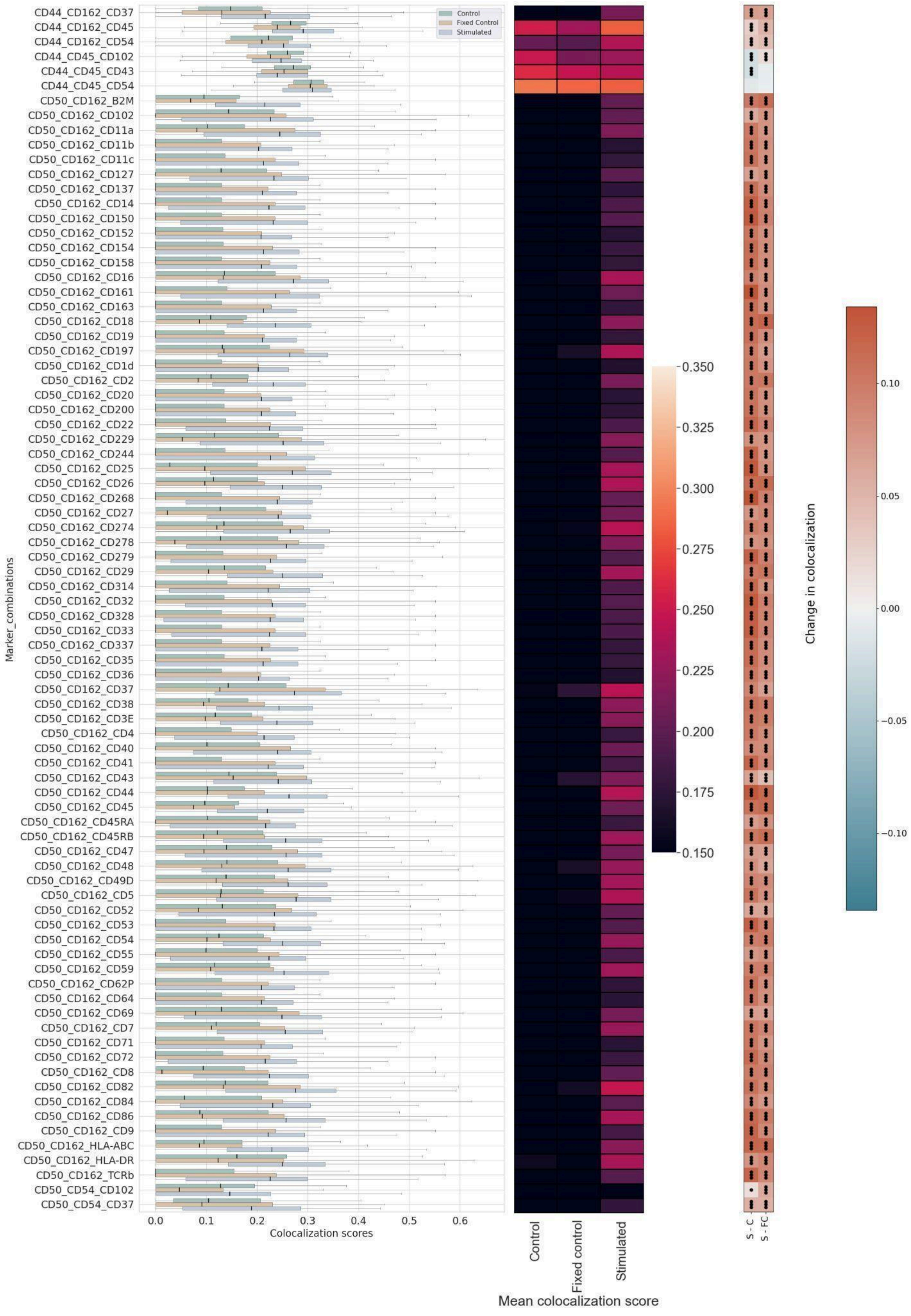

Supplementary Figure 11. Higher order differential colocalization, from the uropod experiment, for the CD50 and CD162 protein pair with all other proteins on the MPX antibody panel. The first column displays the higher order distribution of scores of the selected proteins (CD50, CD162) with each protein in the MPX panel, except controls. The second column shows a heatmap with the mean higher order colocalization in each of the conditions. The last column shows the differential colocalization score when comparing stimulated to both control experiments and their significance. The dots indicate p-value ranges generated by a Wilcoxon rank test: 0 dots (p-value > 0.01), 1 dot (0.001 < p-value <= 0.01), 2 dots (0.0001 < p-value <= 0.001) and 3 dots (p-value <= 0.0001).

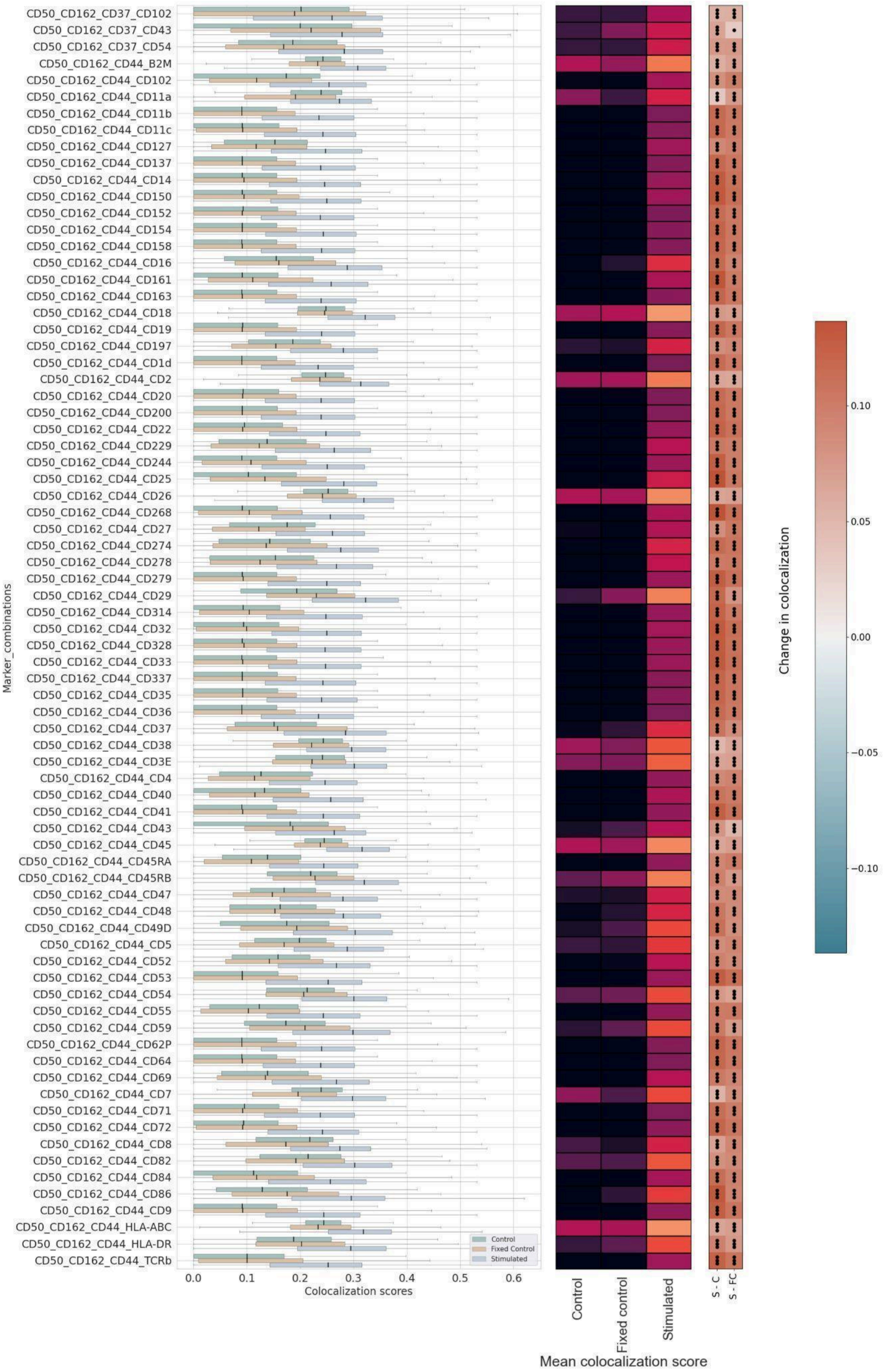
Marker_combinations
CD50_CD162_CD37_CD102
CD50_CD162_CD37_CD43
CD50_CD162_CD37_CD54
CD50_CD162_CD44_B2M
CD50_CD162_CD44_CD102
CD50_CD162_CD44_CD11a
CD50_CD162_CD44_CD11b
CD50_CD162_CD44_CD11c
CD50_CD162_CD44_CD127
CD50_CD162_CD44_CD137
CD50_CD162_CD44_CD14
CD50_CD162_CD44_CD150
CD50_CD162_CD44_CD152
CD50_CD162_CD44_CD154
CD50_CD162_CD44_CD158
CD50_CD162_CD44_CD16
CD50_CD162_CD44_CD161
CD50_CD162_CD44_CD163
CD50_CD162_CD44_CD18
CD50_CD162_CD44_CD19
CD50_CD162_CD44_CD197
CD50_CD162_CD44_CD1d
CD50_CD162_CD44_CD2
CD50_CD162_CD44_CD20
CD50_CD162_CD44_CD200
CD50_CD162_CD44_CD22
CD50_CD162_CD44_CD229
CD50_CD162_CD44_CD244
CD50_CD162_CD44_CD25
CD50_CD162_CD44_CD26
CD50_CD162_CD44_CD268
CD50_CD162_CD44_CD27
CD50_CD162_CD44_CD274
CD50_CD162_CD44_CD278
CD50_CD162_CD44_CD279
CD50_CD162_CD44_CD29
CD50_CD162_CD44_CD314
CD50_CD162_CD44_CD32
CD50_CD162_CD44_CD328
CD50_CD162_CD44_CD33
CD50_CD162_CD44_CD337
CD50_CD162_CD44_CD35
CD50_CD162_CD44_CD36
CD50_CD162_CD44_CD37
CD50_CD162_CD44_CD38
CD50_CD162_CD44_CD3E
CD50_CD162_CD44_CD4
CD50_CD162_CD44_CD40
CD50_CD162_CD44_CD41
CD50_CD162_CD44_CD43
CD50_CD162_CD44_CD45
CD50_CD162_CD44_CD45RA
CD50_CD162_CD44_CD45RB
CD50_CD162_CD44_CD47
CD50_CD162_CD44_CD48
CD50_CD162_CD44_CD49D
CD50_CD162_CD44_CD5
CD50_CD162_CD44_CD52
CD50_CD162_CD44_CD53
CD50_CD162_CD44_CD54
CD50_CD162_CD44_CD55
CD50_CD162_CD44_CD59
CD50_CD162_CD44_CD62P
CD50_CD162_CD44_CD64
CD50_CD162_CD44_CD69
CD50_CD162_CD44_CD7
CD50_CD162_CD44_CD71
CD50_CD162_CD44_CD72
CD50_CD162_CD44_CD8
CD50_CD162_CD44_CD82
CD50_CD162_CD44_CD84
CD50_CD162_CD44_CD86
CD50_CD162_CD44_CD9
CD50_CD162_CD44_HLA-ABC
CD50_CD162_CD44_HLA-DR
CD50_CD162_CD44_TCRb
Control
Fixed Control
Stimulated
0.0
0.1
0.2
0.3
0.4
0.5
0.6
Colocalization scores
Control
Fixed control
Stimulated
Mean colocalization score
S - C
S - FC
0.10
0.05
0.00
−0.05
−0.10
Change in colocalization

Supplementary Figure 12. Higher order differential colocalization, from the uropod experiment, for the CD50, CD44 and CD162 trio with all other markers on the MPX antibody panel. The first column displays the higher order distribution of scores of the selected proteins with all other proteins in the panel, except controls. It also includes a few proteins of reference with the CD50, CD162 and CD37 trio. The second column shows a heatmap with the mean higher order colocalization in each of the conditions. The last column shows the differential colocalization score when comparing stimulated to both control experiments and their significance. The dots indicate p-value ranges generated by a Wilcoxon rank test: 0 dots (p-value > 0.01), 1 dot (0.001 < p-value <= 0.01), 2 dots (0.0001 < p-value <= 0.001) and 3 dots (p-value <= 0.0001).

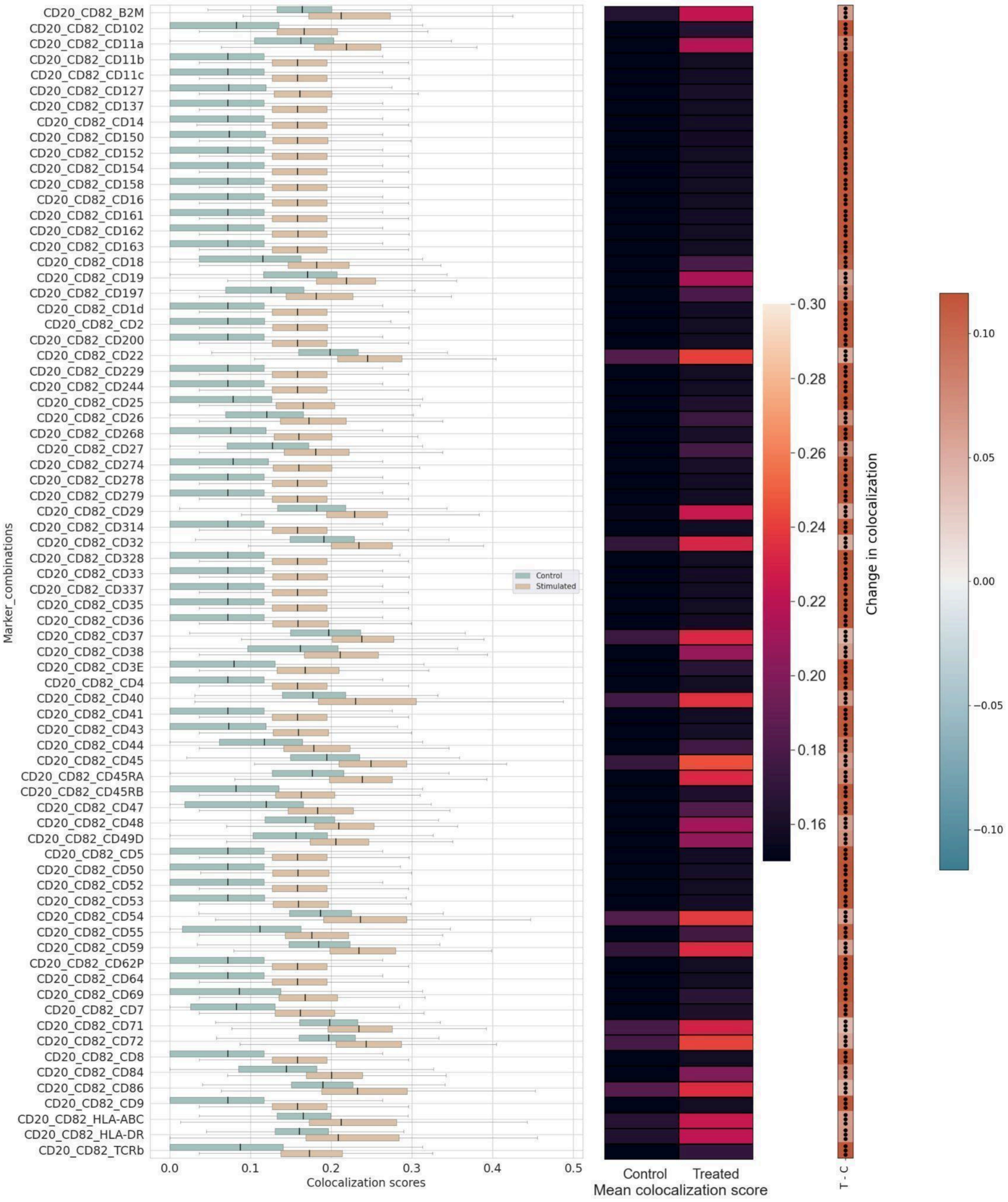
Marker_combinations
Colocalization scores
Control
Stimulated
Control
Treated
Mean colocalization score
T - C
Change in colocalization
CD20_CD82_B2M
CD20_CD82_CD102
CD20_CD82_CD11a
CD20_CD82_CD11b
CD20_CD82_CD11c
CD20_CD82_CD127
CD20_CD82_CD137
CD20_CD82_CD14
CD20_CD82_CD150
CD20_CD82_CD152
CD20_CD82_CD154
CD20_CD82_CD158
CD20_CD82_CD16
CD20_CD82_CD161
CD20_CD82_CD162
CD20_CD82_CD163
CD20_CD82_CD18
CD20_CD82_CD19
CD20_CD82_CD197
CD20_CD82_CD1d
CD20_CD82_CD2
CD20_CD82_CD200
CD20_CD82_CD22
CD20_CD82_CD229
CD20_CD82_CD244
CD20_CD82_CD25
CD20_CD82_CD26
CD20_CD82_CD268
CD20_CD82_CD27
CD20_CD82_CD274
CD20_CD82_CD278
CD20_CD82_CD279
CD20_CD82_CD29
CD20_CD82_CD314
CD20_CD82_CD32
CD20_CD82_CD328
CD20_CD82_CD33
CD20_CD82_CD337
CD20_CD82_CD35
CD20_CD82_CD36
CD20_CD82_CD37
CD20_CD82_CD38
CD20_CD82_CD3E
CD20_CD82_CD4
CD20_CD82_CD40
CD20_CD82_CD41
CD20_CD82_CD43
CD20_CD82_CD44
CD20_CD82_CD45
CD20_CD82_CD45RA
CD20_CD82_CD45RB
CD20_CD82_CD47
CD20_CD82_CD48
CD20_CD82_CD49D
CD20_CD82_CD5
CD20_CD82_CD50
CD20_CD82_CD52
CD20_CD82_CD53
CD20_CD82_CD54
CD20_CD82_CD55
CD20_CD82_CD59
CD20_CD82_CD62P
CD20_CD82_CD64
CD20_CD82_CD69
CD20_CD82_CD7
CD20_CD82_CD71
CD20_CD82_CD72
CD20_CD82_CD8
CD20_CD82_CD84
CD20_CD82_CD86
CD20_CD82_CD9
CD20_CD82_HLA-ABC
CD20_CD82_HLA-DR
CD20_CD82_TCRb
0.0
0.1
0.2
0.3
0.4
0.5
0.30
0.28
0.26
0.24
0.22
0.20
0.18
0.16
0.10
0.05
0.00
−0.05
−0.10

Supplementary Figure 13. Higher order differential colocalization, from the Rituximab experiment, for the CD20 and CD82 pair with all other proteins of the MPX antibody panel. The first column displays the higher order distribution of scores of the selected proteins with all other proteins, excluding controls. The second column shows a heatmap with the mean higher order colocalization in each of the conditions. of order 3 and the last column shows the differential colocalization score when comparing the Rituximab-stimulated cells to the control experiment and their significance. The dots indicate p-value ranges generated by a Wilcoxon rank test: 0 dots (p-value > 0.01), 1 dot (0.001 < p-value <= 0.01), 2 dots (0.0001 < p-value <= 0.001) and 3 dots (p-value <= 0.0001).

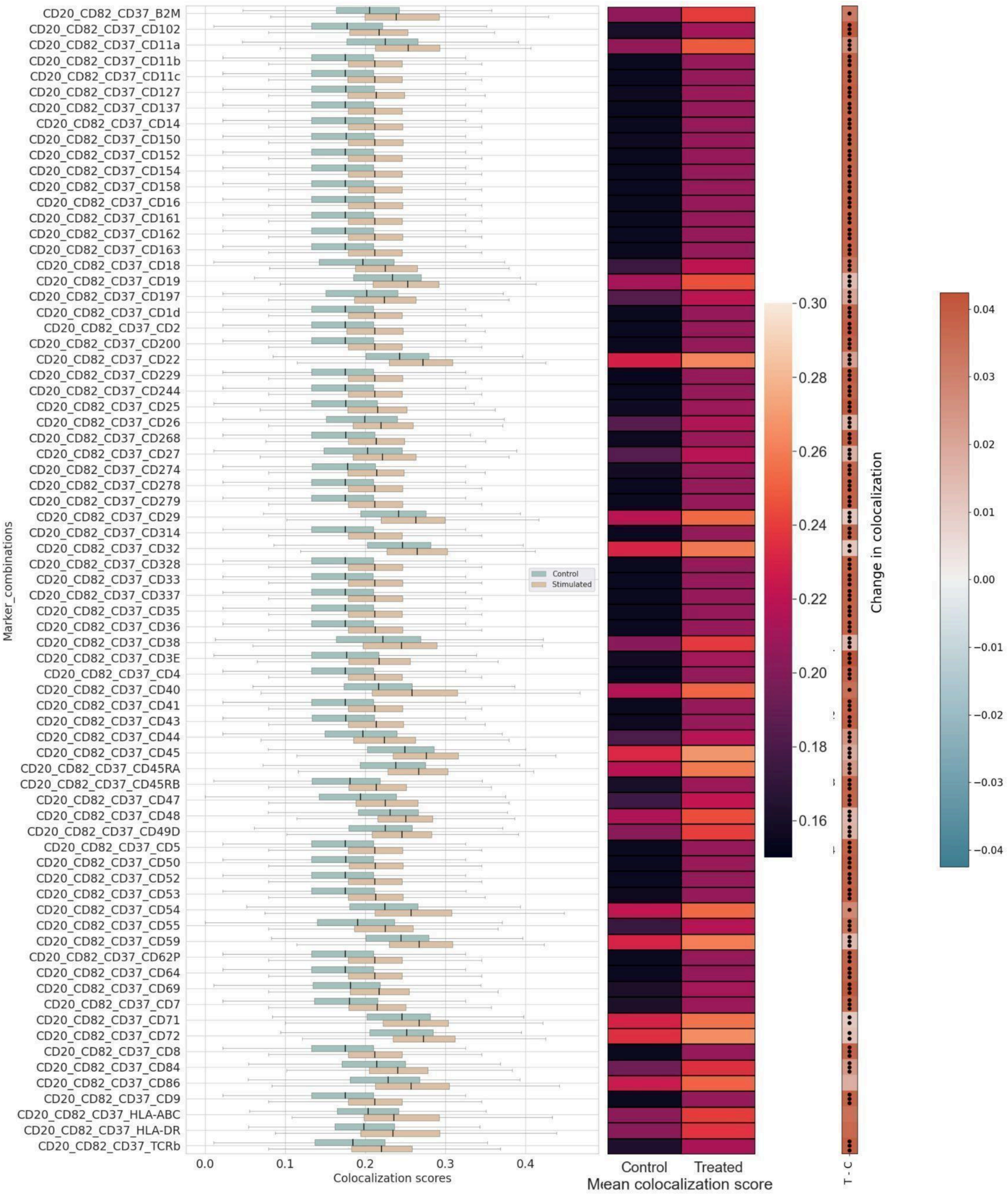

Supplementary Figure 14. Higher order differential colocalization, from the Rituximab experiment, for the CD20, CD82 and CD37 trio with all other markers of the MPX antibody panel. The first

column displays the higher order distribution of scores of the selected proteins with all other proteins, excluding controls. The second column shows a heatmap with the mean higher order colocalization in each of the conditions of order 4 and the last column shows the differential colocalization score when comparing the Rituximab-stimulated cells to the control experiment and their significance. The dots indicate p-value ranges generated by a Wilcoxon rank test: 0 dots (p-value > 0.01), 1 dot (0.001 < p-value <= 0.01), 2 dots (0.0001 < p-value <= 0.001) and 3 dots (p-value <= 0.0001).
